# A multi-omic analysis of MCF10A cells provides a resource for integrative assessment of ligand-mediated molecular and phenotypic responses

**DOI:** 10.1038/s42003-022-03975-9

**Published:** 2022-10-07

**Authors:** Sean M. Gross, Mark A. Dane, Rebecca L. Smith, Kaylyn L. Devlin, Ian C. McLean, Daniel S. Derrick, Caitlin E. Mills, Kartik Subramanian, Alexandra B. London, Denis Torre, John Erol Evangelista, Daniel J. B. Clarke, Zhuorui Xie, Cemal Erdem, Nicholas Lyons, Ted Natoli, Sarah Pessa, Xiaodong Lu, James Mullahoo, Jonathan Li, Miriam Adam, Brook Wassie, Moqing Liu, David F. Kilburn, Tiera A. Liby, Elmar Bucher, Crystal Sanchez-Aguila, Kenneth Daily, Larsson Omberg, Yunguan Wang, Connor Jacobson, Clarence Yapp, Mirra Chung, Dusica Vidovic, Yiling Lu, Stephan Schurer, Albert Lee, Ajay Pillai, Aravind Subramanian, Malvina Papanastasiou, Ernest Fraenkel, Heidi S. Feiler, Gordon B. Mills, Jake D. Jaffe, Avi Ma’ayan, Marc R. Birtwistle, Peter K. Sorger, James E. Korkola, Joe W. Gray, Laura M. Heiser

**Affiliations:** 1 Department of Biomedical Engineering, OHSU, Portland, OR USA; 2 Laboratory of Systems Pharmacology, Department of Systems Biology, Harvard Program in Therapeutic Science, Harvard Medical School, Boston, MA USA; 3 Department of Pharmacological Sciences, Mount Sinai Center for Bioinformatics, Icahn School of Medicine at Mount Sinai, New York, NY USA; 4 Department of Chemical and Biomolecular Engineering, Clemson University, Clemson, SC USA; 5 Broad Institute of MIT and Harvard, Cambridge, MA USA; 6 Department of Biological Engineering, Massachusetts Institute of Technology, Cambridge, MA USA; 7 Sage Bionetworks, Seattle, WA USA; 8 Sylvester Comprehensive Cancer Center, University of Miami, Miami, FL 33136 USA; 9 Department of Molecular and Cellular Pharmacology, Miller School of Medicine, University of Miami, Miami, FL 33136 USA; 10 Institute for Data Science & Computing, University of Miami, Miami, FL 33136 USA; 11 Department of Genomic Medicine, Division of Cancer Medicine, The University of Texas MD Anderson Cancer Center, Houston, TX USA; 12 Heart, Lung, and Blood Institute, National Institutes of Health, Bethesda, USA; 13 Human Genome Research Institute, National Institutes of Health, Bethesda, USA; 14 Knight Cancer Institute, OHSU, Portland, OR USA; 15 Division of Oncological Sciences, OHSU, Portland, OR USA

**Keywords:** Regulatory networks, Cell growth

## Abstract

The phenotype of a cell and its underlying molecular state is strongly influenced by extracellular signals, including growth factors, hormones, and extracellular matrix proteins. While these signals are normally tightly controlled, their dysregulation leads to phenotypic and molecular states associated with diverse diseases. To develop a detailed understanding of the linkage between molecular and phenotypic changes, we generated a comprehensive dataset that catalogs the transcriptional, proteomic, epigenomic and phenotypic responses of MCF10A mammary epithelial cells after exposure to the ligands EGF, HGF, OSM, IFNG, TGFB and BMP2. Systematic assessment of the molecular and cellular phenotypes induced by these ligands comprise the LINCS Microenvironment (ME) perturbation dataset, which has been curated and made publicly available for community-wide analysis and development of novel computational methods (synapse.org/LINCS_MCF10A). In illustrative analyses, we demonstrate how this dataset can be used to discover functionally related molecular features linked to specific cellular phenotypes. Beyond these analyses, this dataset will serve as a resource for the broader scientific community to mine for biological insights, to compare signals carried across distinct molecular modalities, and to develop new computational methods for integrative data analysis.

## Abstract (web summary)

Comprehensive profiling of ligand-induced perturbation responses of the MCF10A mammary epithelial cell line provides an exhaustive resource for identification of the molecular basis of cellular phenotypes.

## Introduction

The function of cells and their organization into tissues is controlled by interactions between cell-intrinsic molecular networks and cell-extrinsic signals, while dysregulation of these signals is associated with various diseases^1^. Extracellular ligands activate cell surface receptors to modulate chromatin, RNA, and protein networks that induce changes in multiple cellular phenotypes including viability^2^, growth rate^3^, motility^4^, polarization, and differentiation state^5^. Disease-specific studies—especially those focused on cancer—have concentrated on understanding phenotypes related to disease progression, resistance mechanisms, therapeutic vulnerabilities, and molecular predictors of response^6–15^. Several canonical signaling pathways have been linked to distinct normal and disease-associated cellular phenotypes, including MAPK^16^, JAK/STAT^17^, WNT^18^, and TGFB^19^. However, a detailed mapping of the linkage between multi-modal molecular and phenotypic responses underlying cell state regulation, developmental processes and diverse diseases is lacking.

Two general approaches have been used to explore the role of extracellular signals in modulating cellular and molecular phenotypes. One approach involves systematic large-scale perturbation of panels of immortalized cell lines, which has yielded libraries of response signatures^6,8–11,13,20–22^. The other approach involves more focused assessment of phenotypic and molecular changes in more complex model systems, including engineered organoids^23,24^, flies^25^, worms^26,27^, fish^28^ and mice^29^. Together these studies indicate that comprehensive multiomic assessment of perturbation responses is critical for gaining insights into molecular-phenotype relationships. From this work, module analysis of multiomic molecular data has proven a powerful approach to identify co-regulated molecular features associated with normal^30–33^ and disease-associated^34^ phenotypes. Such data-driven approaches require comprehensive, systematically-generated datasets, and in recognition of this, multiple data generation consortia have emerged over the past 20 years, including ENCODE^35^, TCGA^36^, GTEx^37^, and HuBMAP^38^.

The Library of Integrated Network-based Cellular Signatures (LINCS) consortium^39^ study presented here is a large-scale, cell line-based perturbation experiment designed to examine the molecular and phenotypic responses of normal cells to perturbations. Its uniqueness lies in the coordinated measurements of many different cellular and molecular responses to biologically relevant ligands that, when studied together, can be used for systems-level analysis of microenvironmental responses. Here we focused on the well-characterized human mammary epithelial MCF10A cell line^40,41^, which is a nontransformed cell line that exhibits many of the key hallmarks of epithelial biology, including migration^42,43^ and organoid formation^44,45^. It is also easily manipulated in a variety of assays including live-cell imaging^46^, knock-down^41^, and chemical perturbation^47^, and therefore is commonly used for cell biology studies. The combination of molecular and cellular properties, as well as its wide adoption in the biomedical research community, provided the rationale for using MCF10A in these studies. Importantly, the focus on a single cell line provided a controlled cell-intrinsic genetic context, which afforded molecular and temporal density in experimental measurements and assessment of multiple perturbations across a variety of assays. We studied responses to six ligands that activate different canonical signaling pathways of biological and clinical relevance, enabling comparison of distinct molecular and phenotypic effects. These data are publicly available for community study at synapse.org/LINCS_MCF10A. The following sections describe and evaluate the information content of the LINCS ME perturbation dataset and present illustrative analyses showing how the dataset can be used to (a) elucidate molecular and cellular phenotypes that are influenced by the binding of specific ligands, (b) identify ligand-induced signatures that can be mined for biological insights, (c) discover candidate causal or functional relationships between molecular features with module analysis, and (d) identify molecular programs that control specific cellular phenotypes.

## Results

### Approach to generate a LINCS ME perturbation dataset

**Fig. 1:**
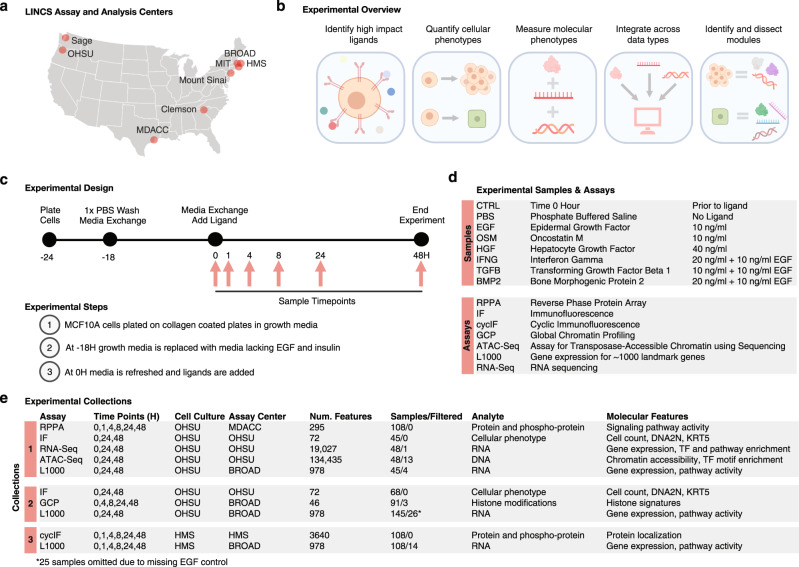
Overview of experimental approach to assess the impact of microenvironmental factors. **a** Map of LINCS data generation and analysis centers. **b** Schematic illustrating the experimental and analytical approaches to link molecular and cellular phenotypes. **c** Schematic of the experimental design, cell culture protocol, and sample harvest time points. **d** The experimental treatments, dosages, and assays deployed to generate the LINCS ME perturbation datasets. **e** Summary of the assays, time points, and features for the three experimental collections.

Eight laboratories supported by the NIH LINCS program contributed to the creation and analysis of an MCF10A microenvironment (ME) perturbation dataset to enable community study of the molecular mechanisms engaged by microenvironmental signals to modulate specific cellular phenotypes (Fig. 1a). Figure 1b shows the experimental and computational steps involved in the creation of the database. The process began with screening and selection of ligands that strongly modulated phenotype. Both phenotypic and molecular responses to ligands were then measured over time and integrated computationally to identify the phenotypes and molecular modules engaged by each ligand. Figure 1c shows the experimental design in which multiple endpoints were measured at several time points after the introduction of ligands. The ligands and experimental assays are summarized in Fig. 1d.

The elucidation of phenotype-associated molecular networks requires study of multiple ligands that modulate cell behaviors through varied signaling pathways. To identify a panel of high-impact ligands, we performed two high-throughput microenvironment microarray (MEMA) screens of 3024 combinations of 63 soluble ligands and 48 insoluble extracellular matrix proteins^48^; one screen with and another without EGF, a typical component of MCF10A growth medium^40^. We focused on collagen-1 as the insoluble extracellular matrix component and identified EGF, HGF, and OSM as ligands that increased growth in the absence of EGF, while BMP2, IFNG, TGFB decreased growth in the presence of EGF (Supplementary Fig. 1a, b). These ligands target highly expressed receptors that are members of different canonical receptor classes (Supplementary Fig. 1c). Dose-response experiments identified the ligand doses necessary to yield maximal changes in cell numbers (Supplementary Fig. 1d and e). Inclusion of EGF in combination with BMP2, IFNG, and TGFB ensured sufficient cell numbers for molecular profiling.

The participating LINCS consortium laboratories performed systematic and large-scale analyses of epigenomic, transcriptomic, proteomic and phenotypic responses to each ligand at several time points during a 48H period after treatment (Fig. 1b, d, and e). Experiments were carefully planned to minimize technical artifacts that are sometimes associated with large-scale experiments, such as cell line drift, variation in reagents, and protocol differences; a detailed description of considerations can be found in Methods. Cells for all analyses were grown and treated at OHSU and the treated cells or lysates were distributed to participating laboratories for analyses, except for those analyzed using cyclic immunofluorescence (CyCIF)^49,50^. Cells for CyCIF were grown and treated at HMS using cells, culture media and ligands supplied by one laboratory at OHSU to minimize experimental variation^51^ (Fig. 1e). For each assay, MCF10A cells were plated on collagen-1-coated cell culture dishes in their standard growth medium, which contains the growth factors EGF and insulin^40^. After attachment, the growth medium was replaced with medium lacking EGF and insulin, and cells were then treated with the ligand panel at optimized concentrations (Fig. 1d).

Samples were collected before and after treatment over the 48H time period beginning with a time 0H sample (referred to as control: CTRL, Fig. 1d). Cellular responses were measured using live-cell imaging, four-color fluorescence imaging and CyCIF^49,50^. Molecular responses were assessed for changes in protein expression with reverse phase protein arrays (RPPA);^52^ chromatin profiling using an Assay for Transposase-Accessible Chromatin using sequencing (ATACseq) and global chromatin profiling (GCP);^53^ RNA expression using RNAseq and the L1000^20^ transcriptomics panel designed to assess the levels of 1000 RNA transcripts. Samples for the different assays were collected in three experimental collections of at least three biological replicates each (Fig. 1e). Logistical and cost constraints resulted in some assays being applied to only a subset of time points. Rigorous quality assessment (see Methods) of all data led to the elimination of ~5% of samples (44/814). The resultant data and metadata are available at: synapse.org/LINCS_MCF10A.

### Overview of the ligand-induced cellular and molecular responses that comprise the LINCS ME perturbation dataset

#### Cellular responses

**Fig. 2:**
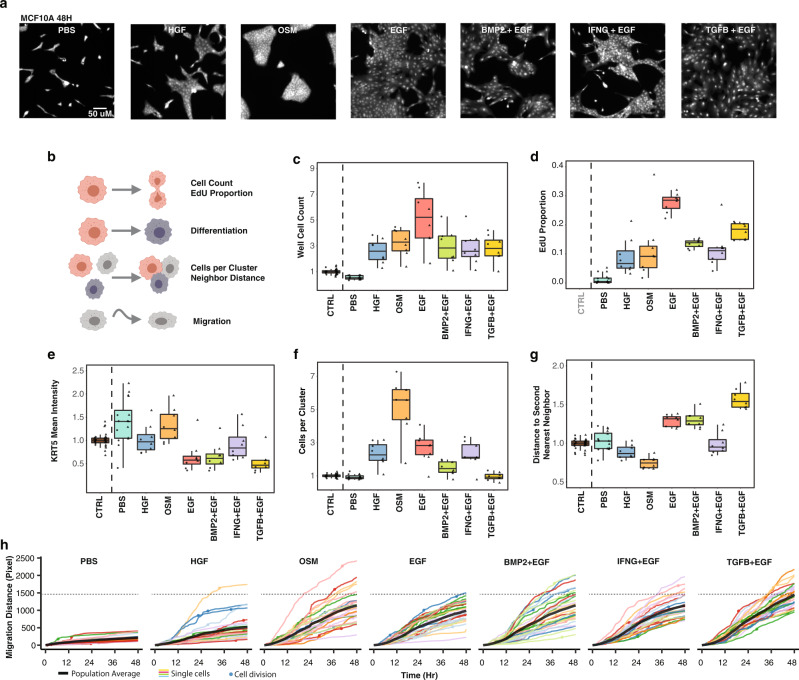
Ligand treatments induce diverse phenotypic responses. **a** Representative immunofluorescent images of ligand-induced cellular phenotypes at 48H. MCF10A cells were stained with Cell Mask to visualize cytoplasm. **b** Cartoon showing the image-based cellular phenotypes assessed from the immunofluorescence and live cell imaging assays. **c**–**g** Boxplots summarizing cellular phenotypes at time 0H (CTRL) and 48H after ligand addition from 8 biological replicates. Individual datapoints represent well-level means normalized to 0H. Circles are from collection 1 and triangles are from collection 2. The interquartile range is indicated by the box, with whiskers extending to no further than 1.5 times the interquartile range. Note that EdU positive proportion was not measured at 0H. Data in Supplementary Data 1. **h** Accumulated cell migration (colored lines) from 0-48H for 25 cell lineages (individual cells and one of their progeny if they divided). Circles indicate mitotic events. The solid black lines indicate the population average; the dotted gray line shows the average TGFB + EGF induced migration at 48H, which was the treatment that induced the greatest increase in cell migration. Data in Supplementary Data 2, 3.

We quantified four-color immunofluorescence images from cells 24H and 48H after ligand treatment to assess cell clustering, cell density, shape, DNA content, and expression of proteins related to differentiation state, which revealed a constellation of changes following each treatment that were quantified with image analysis (Fig. 2a, b and Supplementary Data 1). CyCIF collected at all time points revealed additional changes in cell state and pathway activity. Consistent with our MEMA screen, HGF, OSM and EGF increased cell numbers and EdU incorporation (a measure of proliferation). BMP2 and TGFB significantly suppressed growth relative to the EGF condition; IFNG also reduced growth (Fig. 2c, d and Supplementary Data 1). HGF, OSM, and IFNG + EGF upregulated KRT5 expression, a marker of basal differentiation state in mammary epithelial cells^54^ (Fig. 2e and Supplementary Data 1). OSM caused cells to form tight clusters (Fig. 2f and Supplementary Data 1). Lastly, TGFB + EGF induced evenly distributed cells with increased size, quantified as an increase in the distance to neighboring cells (Fig. 2g and Supplementary Data 1). Together, these ligands constitute a powerful set of perturbations to probe molecular and phenotypic networks.

Analysis of live-cell images showed the emergence of each phenotype following ligand treatment (Supplementary Movies 1–7**)**. OSM induced cells to undergo collective migration, a unique phenotype among the tested ligands. We assessed cell migration by tracking individual cells across the 48 hour time period and quantified migration as the total distance traversed by each cell lineage (Fig. 2h and Supplementary Data 2, 3). In all ligand conditions, cell migration increased compared to the PBS condition, but to varying degrees: HGF-treated cells migrated the least while TGFB + EGF induced the greatest migration (Tukey’s HSD, p-value < 9×10^−7^). Together, the live cell imaging and migration analyses show the dynamic emergence of distinct phenotypic responses by each of the ligand treatments.

#### Molecular responses

**Fig. 3:**
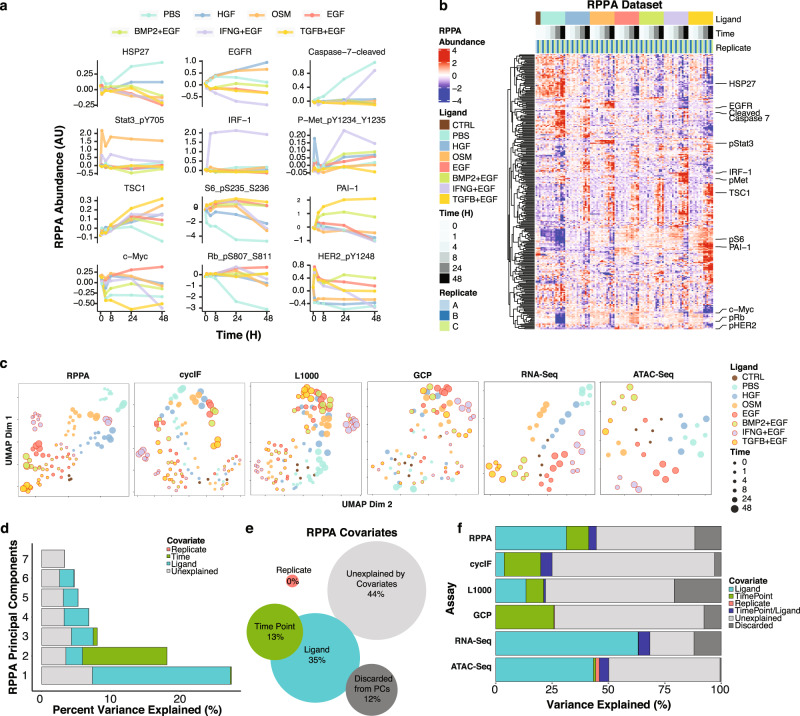
Six molecular assays reveal diverse dynamic responses to treatments. **a** Line graphs show dynamic responses for 12 proteins measured in the RPPA assay under the different ligand treatments. **b** Heatmap of protein abundances as measured by RPPA. Rows represent abundance of 295 (phosphor)proteins and are median-centered and hierarchically clustered. Columns represent individual replicate samples, ordered by treatment and time. Callouts show the 12 proteins from panel A. **c** UMAPs for each of the six molecular assays. Each dot represents data from an individual sample and is the 2-dimensional embedding of all features measured in the assay. Color indicates ligand treatment and size indicates time point. **d** Plot of the first two principal components (PCs) of RPPA assay. Variance in PC1 and PC2 is largely driven by ligand treatment and experimental time point, respectively. Data in Supplementary Data 6. **e** Analysis of RPPA covariates reveals the proportion of variance explained by sample replicate, experimental time point, and ligand treatment for each of the top seven principal components of the RPPA dataset. (f) Stacked bar graph shows a comparison of the information content contained within each molecular assay. Data in Supplementary Data 7.

The responses to ligands involved numerous features in each of the molecular datasets. Here we demonstrate some of our key observations through analysis of the RPPA proteomic dataset as an exemplar use-case. We assessed the modulation of canonical signaling proteins downstream from each ligand (Fig. 3a and Supplementary Data 4). These included: IRF1, a transcriptional target of STAT1 downstream of IFNG; pSTAT3, a signaling pathway component for OSM; and phosphorylation of MET, the receptor for HGF. PAI-1 provided an assessment of SMAD transcriptional activity, which is downstream of TGFB and BMP2. Additionally, phospho- HER2 provided a readout for conditions that contained EGF in the media. Each of these features were modulated as expected based on prior literature, validating the robustness of the dataset.

Unsupervised hierarchical clustering of the RPPA data set revealed dynamic changes in the protein landscape over time, with some responses shared by multiple ligands and others that were uniquely induced (Fig. 3b and Supplementary Data 5). All treatments that included EGF induced proteins related to growth factor signaling (e.g. pS6). The PBS condition, which lacks added growth factors, showed protein changes associated with reduced proliferation (e.g. decreased pRB) and induction of apoptosis (e.g. cleaved caspase 7), indicating that absence of growth factor signals strongly modulates phenotypic and molecular state.

To gain a high-level view of the six molecular assays, we performed Uniform Manifold Approximation and Projection (UMAP)^55^ dimensionality reduction for all ligand-induced responses (Fig. 3c). Most assays showed ligand-specific effects, as observed by samples from the same ligand treatment tending to group near one another. In addition, most datasets showed evolution over time from the starting state to another distinct state, captured by early time points clustering near the center of the UMAP and later time points for each ligand appearing in different UMAP regions. Principal Component Analysis revealed similar findings, though the variance was manifest in multiple components.

#### Assessment of assay variance

We applied the Measuring Association between VaRIance and Covariates method to systematically assess the fractional variance explained by ligand, time, and replicate^56,57^. In brief, we first performed principal component analysis to reduce the dimensionality of each data set while preserving the variability. Next, we quantified the total variance explained by each covariate (ligand, time, replicate) by summing the weighted variances of all statistically significant principal components (PCs). For example, in the RPPA dataset, the signal in the first PC was dominated by ligand while the second PC was dominated by time point (Fig. 3d and Supplementary Data 6). We reasoned that PCs with an eigenvalue of less than 0.7 were unlikely to significantly correlate to any covariates and discarded these from the analysis. Summing across all significant PCs from the RPPA dataset revealed that 35% of the variance could be attributed to ligand and 13% to time point (Fig. 3e and Supplementary Data 6). Variance explained by multiple co-variates is represented by overlap in the Venn diagram. Overall, 44% of the variance in the RPPA dataset could not be explained by one of these factors, suggesting signal in the data attributable to other factors, such as changes shared by multiple ligands. Similarly, all other assays carried signal attributable to ligand treatment, although to varying degrees: RNAseq (63.1%) and ATACseq (43.3%) contained the greatest ligand-associated signal while GCP (0.1%) contained the least (Fig. 3f and Supplementary Data 7). Datasets with both early and late time points (RPPA, GCP, CyCIF) carried signal attributable to time. There was limited variation attributable to replicates across all assays, indicating modest biological and technical variation.

### Identification and analysis of ligand-induced molecular signatures

Here we present a systematic assessment of molecular signatures induced by each ligand and provide examples of how these signatures can be analyzed and mined. Specifically, we focus on IFNG + EGF to examine the temporal evolution of responses across modalities and to identify immune-related molecular features.

#### Identification of ligand-induced signatures

**Fig. 4:**
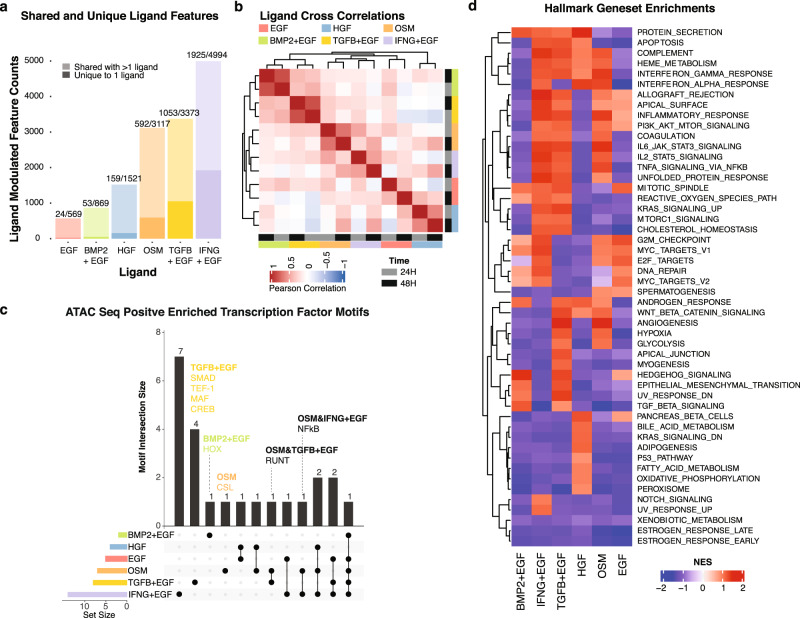
Assessment of ligand-induced molecular change. **a** Barplot showing the number of features significantly modulated by each ligand treatment at 24H or 48H. Shading indicates whether induced features are unique to a particular treatment (dark) or induced by multiple treatments (light). Numbers above bars indicate the number of features uniquely induced over the total number of features induced. Data in Supplementary Data 8. **b** Heatmap showing pairwise correlations between molecular features induced by each ligand. Ligand responses from similar families are more highly correlated than those from unrelated families. **c** UpSet plot showing overlaps of induced transcription factor motifs among ligand treatments calculated from ATACseq data at 24H or 48H. Column heights represent the number of transcription factor motifs induced by the ligand(s) indicated with filled dots. Data in Supplementary Data 12. **d** Hallmark Geneset enrichment scores computed from RNAseq data at 24H.

To create molecular signatures of ligand responses, we identified features from each of the 6 data types that were differentially expressed at 24H and 48H time points relative to the CTRL sample (q-value <0.01, |logFC | ≥ 1.5) (Fig. 4a and Supplementary Data 8). Features were classified as unique if they were modulated by a single treatment or shared if they were induced by more than one treatment (Supplementary Data 9 and 10). All treatments induced both unique and shared molecular responses. IFNG + EGF, TGFB + EGF and OSM induced the greatest shift in molecular state, as measured by the total number of features induced across the RNAseq, ATACseq, GCP, CyCIF and RPPA assays. In contrast, EGF, HGF and BMP2 + EGF showed more modest effects, consistent with maintenance of MCF10A cells in a pre-treated state. Cross-correlation analysis of the molecular responses revealed that 24H and 48H responses were strongly correlated for each ligand and that responses to ligands from related families (BMP2/TGFB, OSM/IFNG, EGF/HGF) were more similar to one another than to other family classes (Fig. 4b and Supplementary Data 11).

Motivated by our observation that the ATACseq and RNAseq datasets carried the strongest ligand signals, we more deeply interrogated these responses. We analyzed ATACseq transcription factor binding motif enrichment, a measure of transcription factor activity, and found that IFNG + EGF and TGFB + EGF induced the greatest number of enriched motifs. For example, TGFB + EGF induced SMAD, TEF-1, MAF and CREB motifs, while TGFB + EGF and OSM both induced changes in RUNT (Fig. 4c and Supplementary Data 12). Gene set enrichment (GSEA) analysis^58^ of the RNAseq dataset revealed a unique complement of gene programs associated with response to each ligand treatment (Fig. 4d and Supplementary Data 13).

Ligand signatures that are strongly anti-correlated with drug-induced transcriptional signatures suggest environmental conditions that a therapeutic inhibitor could reverse and therefore may serve as a sensitizing signal, for example by inhibiting a ligand-activated pathway. Alternatively, if a ligand activates a pathway not affected by drug, this could serve as a possible bypass pathway to mediate resistance, which is captured as non-correlated responses. To test this, we compared our ligand signatures against the LINCS L1000 database^59^ of drug and other chemical response signatures (Fisher exact test, q-value<0.2). While some therapeutic inhibitor signatures were correlated with multiple ligands, the responses to most ligands were associated with a unique complement of inhibitor signatures (Supplementary Fig. 2 and Supplementary Data 14). For example, TGFB + EGF, BMP2 + EGF, and EGF were negatively correlated with SRC inhibition, indicating that these ligands induce similar pathway activation along the SRC signaling axis. EGFR/JAK inhibitors were negatively correlated with OSM, suggesting that cells grown in OSM-rich environments may be particularly sensitive to JAK inhibition. All together, these findings indicate that extracellular ligands activate some of the same molecular programs as therapeutic inhibitors and that the impact of environmental signals on cellular and molecular state is an important consideration for identification of effective therapeutic regimens.

#### Identification of molecular features induced by IFNG

We analyzed responses to IFNG + EGF to illustrate how the LINCS ME perturbation dataset can be used to study the molecular mechanisms associated with ligand responses across time. IFNG is a soluble cytokine secreted by cells of both the innate and adaptive immune systems and has become increasingly scrutinized, owing to interest in understanding the role of the immune system in diverse pathophysiologies^60^ as well as cancer immunotherapies. IFNG + EGF treatment induced dynamic changes in canonical IFNG signaling molecules measured across assays and time, including: rapid nuclear translocation of STAT1, the resultant induction of IRF1 followed by upregulation of PDL1 at the membrane as well as associated epigenetic changes (Supplementary Fig. 3a–f). These findings indicate that the LINCS ME perturbation dataset enables the encoding of a stimulus to be traced across time and molecular modalities.

We observed that 66/202 Pathcards Reactome IFNG superpathway features^61^ were among the most strongly modulated by IFNG + EGF treatment, indicating the induction of multiple known signaling responses (Supplementary Fig. 3g). To gain deeper insight into the ability of IFNG to influence both adaptive and innate immune responses through altering cytokine production by malignant cells, we compared the MCF10A IFNG + EGF signature, the IFNG superpathway, and a curated cytokine gene list^62^. This comparison identified 15 cytokines not already included in the IFNG superpathway, suggesting additional cytokines produced by malignant cells in response to IFNG that may interact with various immune cell subsets, including: CSF1^63,64^, IL15^65^, IL12A^66^, CCL2^67^, and CXCL2^68^. This demonstrates how the LINCS ME dataset can be mined to gain biological insights into immune-related signaling and to prioritize molecular features for future study.

### Discovery of candidate functional relationships between molecular features

We reasoned that the patterns of robust multi-omic molecular changes induced across the panel of ligands could be analyzed together to discover coordinately regulated molecular programs. Importantly, our use of multiple ligands that perturb cells along various phenotypic and molecular axes enabled distinct molecular programs to be disentangled. Below we summarize our assessment of the relationships between different modalities, our approach to identify coordinately regulated biological modules, and also illustrate the utility of the modules to provide insights into the molecular programs active across diverse tissues.

#### Identification of coordinately regulated modules

We assessed coordinated responses in the RPPA, RNAseq, L1000, and ATACseq datasets by comparing molecular cognates across datasets that could be mapped through gene names (e.g. Cyclin B1 in RPPA and *CCNB1* in RNAseq). This revealed broad concordance, indicating conserved responses across molecular modalities (Supplementary Fig. 4). For example, the relationships between RPPA and RNAseq showed several patterns: linear correlation (*CCNB1*, *DUSP4*); ligand-specific effects (*PDL1*, *JAK2*); or no association, which typically reflected only modest ligand-induced changes in abundance (*RPS6*, *RB1*). We assessed response concordance, which we defined as similar induction (up- or down-regulation) as compared to the CTRL samples, which revealed 40/207 features were concordantly up-regulated and 30/187 features were concordantly down-regulated in the RNAseq and RPPA datasets. Importantly, we also observed that 2717/3035 features were concordantly unchanged. Next, we measured Pearson correlation of RNAseq and L1000 gene expression measurements for matched and unmatched samples and found that matched samples were on average significantly better correlated than gene expression profiles from unmatched samples (Mann-Whitney U test; p < 2.2*10^−16^, Supplementary Fig. 4d). In a third cross assay comparison, we found that chromatin accessibility was bimodal and that promoter accessibility was associated with transcriptional expression, consistent with prior studies^69^ (Supplementary Fig. 4e). Finally, we compared the 10 most-variant ATACseq transcription factor motifs to single-sample gene set enrichment scores for the same transcription factors from CyCIF data and found that they were generally concordant (Supplementary Fig. 4f).

We next used a systematic approach to identify modules comprised of coordinately regulated molecular features measured in the different assays (CyCIF, RPPA, GCP, and RNAseq, and ATACseq). Specifically, we examined all molecular features that were induced by at least one ligand (see Fig. 4a) and then scaled each assay dataset with rrscale, which is a transformation that normalizes feature distributions, removes outliers, and z-scales feature values^70^ (Supplementary Fig. 5). We used gap analysis^71^ to identify the optimal number of clusters, and then used consensus clustering with partitioning around medoids (PAM) to identify stable clusters. To further ensure that the clusters represented unique expression patterns, we calculated their pairwise correlations and combined highly correlated pairs, which yielded a final set of 14 molecular modules for interpretation (Supplementary Fig. 6a–c).

**Fig. 5:**
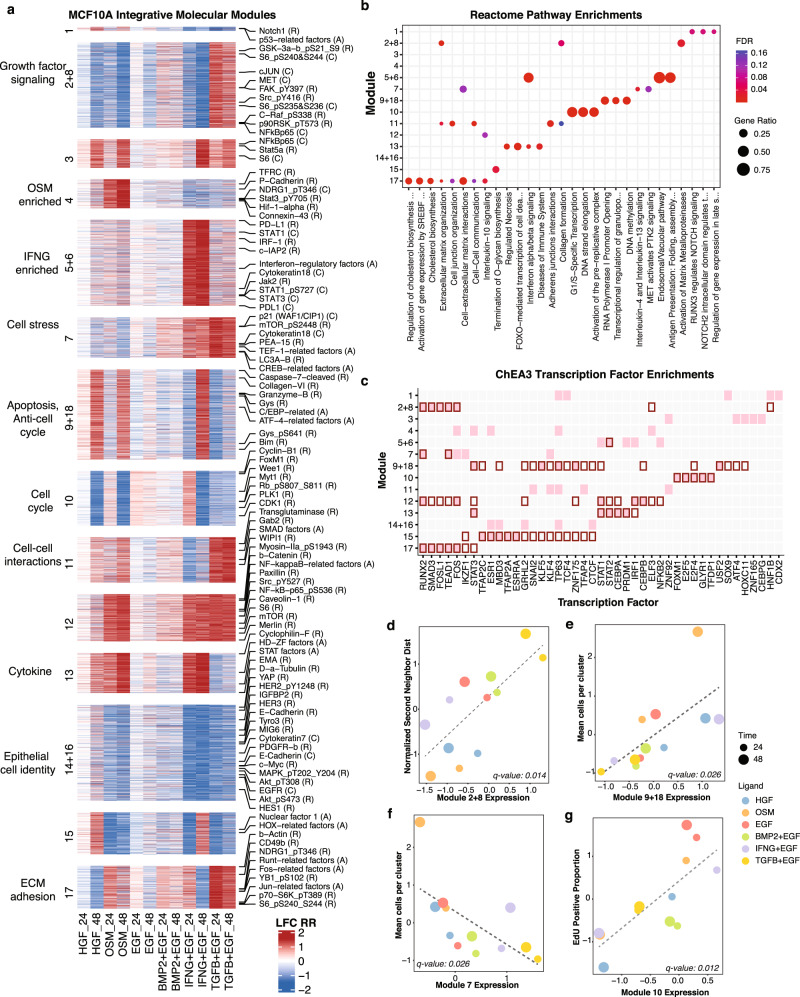
Integrated analysis identifies co-regulated molecular modules. **a** Heatmap showing the 14 integrative molecular modules for each ligand at 24H and 48H. Features are grouped by cluster. Biological interpretation for modules is indicated on the left; feature callouts for RPPA (R), CyCIF (C), ATACseq (A) are shown to the right. **b** Bubble plot shows the top enriched Reactome pathways in each module, computed from RNAseq features. Dot size indicates the gene ratio; dot color indicates FDR value. **c** Heatmap showing the five top-ranked ChEA3 transcription factor enrichments computed from the RNAseq features in each module (pink). Red border indicates transcription factor enrichments with a q-value below 0.2 (FDR-adjusted Fisher’s exact test). **d**–**g** Scatterplots show the relationships between module activity and quantitative phenotypic responses for selected pairs. Dot color indicates the ligand treatment and dot size indicates the time point. The black dotted line shows the linear fit, and the q-value of the fit is shown at the bottom of the plot.

Each module represents a unique complement of co-regulated proteomic, transcriptional, and chromatin features (Fig. 5a and Supplementary Data 15). Features from each assay were distributed across modules, indicating that our analytical approach enabled integration of features measured in diverse assays (Supplementary Fig. 6d). Each module showed distinct modulation patterns across the ligands; most modules were induced by more than one ligand while a few were ligand-specific, consistent with the findings in Fig. 4. Reactome pathway enrichment analysis demonstrated that each module induced an array of transcriptional programs (Fig. 5b and Supplementary Data 16). Transcription Factor enrichment via ChEA3^72^ identified key molecular drivers associated with these modules (Fig. 5c and Supplementary Data 17). To explore how our clustering method compared against other published multiomics approaches^73^, we performed a Consensus Principal Component Analysis (CPCA) using the R package MoCluster^74^, which showed similar ligand-specific expression patterns (Supplementary Fig. 6e–i).

#### Assessment of molecular modules across diverse tissues

Elucidating the molecular programs operable across different tissue types is critical for understanding normal organ development and function and for identifying molecular programs that may go awry in the case of disease. We assessed RNA expression of the 14 integrated modules in the GTEx non-diseased tissue dataset^37^ to identify molecular programs that may be most active in particular tissue types (Supplementary Fig. 7 and Supplementary Data 18). We observed tissue-specific activation of the modules. For example, Module 14 + 16 included features associated with epithelial cell identity such as cytokeratin-7, E-cadherin, claudin-7, and EGFR, and was upregulated in vagina, esophagus, and skin. These tissues are comprised principally of stratified squamous epithelial cells which undergo rapid terminal differentiation as they migrate from a basal zone to cornified surfaces^75–77^. This suggests that deeper analysis of the molecular features coordinately regulated by module 14 + 16 may shed light on key molecular programs important for differentiation and maintenance of epithelial cell state across diverse tissues. Module 2 + 8 was enriched in extracellular matrix organization and collagen formation pathways. This module was highly expressed in artery samples, consistent with the observation that the arterial wall produces a rich and complex extracellular matrix that defines the mechanical properties of the vessel^78,79^. Additional features included in each of these modules may provide additional insights into their roles in normal and diseased processes in different tissues.

### Investigation of the relationship between molecular modules and cellular phenotype

Elucidation of the molecular mechanisms that control cellular phenotype remains a difficult problem in systems biology. We illustrate here how the LINCS ME perturbation dataset can be analyzed to gain insights into mechanisms of phenotype control by linking cellular and molecular responses. We present two examples: a data-driven discovery of associations between phenotypic responses and module activity, followed by a detailed analysis of Module 4 to uncover molecular features associated with the cell clustering and collective motility phenotype induced by OSM.

#### Data-driven discovery of phenotype-module associations

We performed correlation analysis to identify molecular modules that were significantly associated with cellular phenotypes measured by imaging (Fig. 5d–g and Supplementary Data 15). For example, Module 2 + 8 was positively correlated with ‘Normalized Second Neighbor Distance’, a metric that reflects both cell size and cell-cell spatial organization (Fig. 5d, *p* value = 0.014). Several features of this module suggest molecular correlates of this phenotypic response, including pathway enrichments in Extracellular matrix organization and Collagen formation. Additionally, the transcription factor *RUNX2*, which was enriched in this module, has been implicated in modulating cell morphology and cell spreading^80^.

**Fig. 6:**
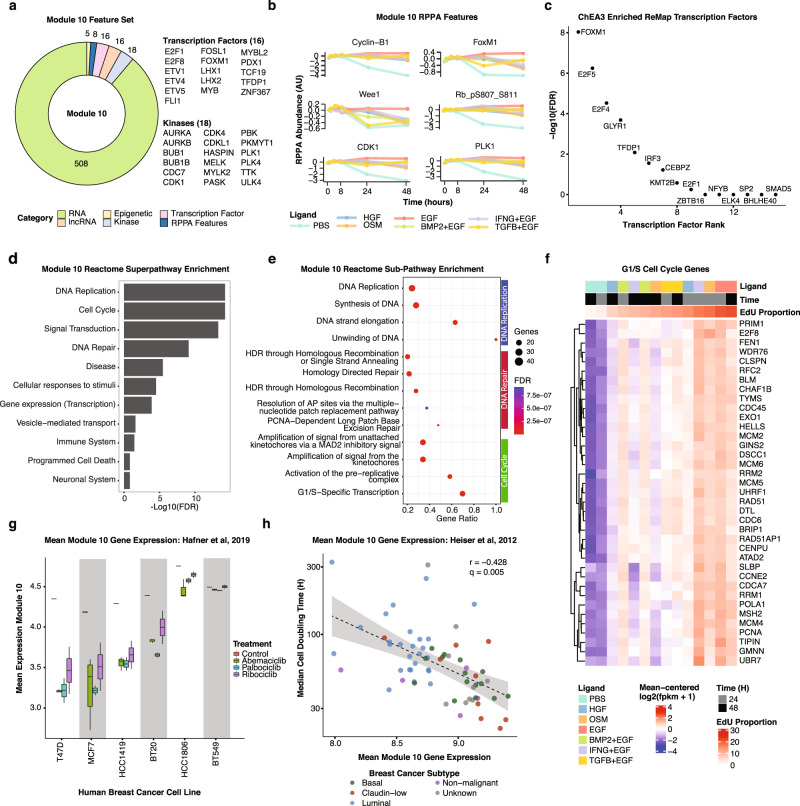
Module 10 is associated with cell cycle progression. **a** Donut plot showing distribution of Module 10 features across assays. Transcription factors and kinases in the RNA gene set are called out to the right of the plot. **b** Line plot showing 6 of the Module 10 RPPA features. Data in Supplementary Data 5. **c** Plot of the top 10 most significantly enriched transcription factors inferred from the Module 10 RNAseq gene set. Data in Supplementary Data 17. **d** Bar plot shows the enrichment of Reactome superpathways from the Module 10 RNA gene set. Data in Supplementary Data 16. **e** Bubble plot showing the top 5 enriched Reactome subpathways from the Reactome Cell Cycle, DNA Repair, and DNA Replication superpathways. Dot color indicates q-value; dot size indicates the number of genes in Module 10 that are found in each gene set. **f** Heat map showing expression of Seurat G1/S cell cycle genes in Module 10 (37 of 43 genes shared), sorted based on the EdU positive proportion. **g** Boxplot of mean Module 10 gene expression for a panel of breast cancer cell lines treated with three CDK4/6 inhibitors for 24H or an untreated control. Cell lines are ordered by abemaciclib GR50 (increasing). The interquartile range is indicated by the box, with whiskers extending to the minimum and maximum values. Data from Hafner, et al.^87^. **h** Dot plot of mean Module 10 gene expression from 65 human breast cancer cell lines graphed against their mean doubling time. Cell lines are colored based on their breast cancer subtype classification. The line indicates the linear fit across all cell lines, with the 95% confidence interval represented by the gray shaded area. Data from Heiser et al.^10^. Figure data in Supplementary Data 15.

We also identified a specific and robust correlation between Module 10 expression and the fraction of EdU positive cells, a measure of cell proliferation (Fig. 5g, *p* value = 0.012). To explore the putative regulatory components of Module 10, we annotated genes that code for transcription factors, kinases, non-coding RNA, and epigenetic regulators (Fig. 6a and Supplementary Data 19). This analysis revealed a suite of factors previously shown to play key roles in regulating cell cycle progression, including the transcription factors: *E2F1, FOXM1, MYB*, and *TFDP1*; and the kinases: *AURKA, CDK1, PLK1*, and *BUB1*. Module 10 RPPA features cyclin B, Wee1, and phosphorylated RB are canonical cell cycle proteins that showed temporal dynamics consistent with changes in proliferation, as well as lesser linked features including FOSL1^81–83^ and PASK^84,85^ (Fig. 6b and Supplementary Data 4). ChEA3 transcription factor enrichment^72^ identified multiple cell cycle-associated transcription factors including *FOXM1*, *TFDP1* and *E2F* isoforms (Fig. 6c and Supplementary Data 17). Among the most significantly enriched Reactome pathways were Cell Cycle, DNA replication, and DNA repair (Fig. 6d and Supplementary Data 16). We analyzed the top 5 subpathways within each of these Reactome pathways and found the highest enrichment for G1/S specific transcription, PCNA-dependent base excision repair, and unwinding of DNA (Fig. 6e and Supplementary Data 16). Additionally, Module 10 included 86% (37/43) of the genes in a functionally-annotated G1/S gene set^86^, with expression patterns consistent with changes in EdU incorporation (Fig. 6f). There is also evidence for DNA damage and potentially for replication stress in the induction base-excision repair, the G2M checkpoint and activation of DNA damage checkpoint associated kinases. In sum, Module 10 contains cell cycle-associated molecular features from multiple modalities.

To test if the link between Module 10 and cell cycle control generalized beyond MCF10A cells, we analyzed two publicly available independently generated breast cancer cell line data sets. First, we quantified mean Module 10 gene expression scores from 7 breast cancer cell lines treated for 24 hours with a panel of CDK4/6 inhibitors^87^. As expected, this showed robust down-regulation of Module 10 in response to each of the three CDK4/6 inhibitors in the five sensitive cell lines, while the two resistant cell lines showed only modest changes in Module 10 expression (Mann-Whitney U test, *p*-value = 0.028, Fig. 6g and Supplementary Data 15). In a second analysis, we compared Module 10 expression for a panel of 65 breast cancer cell lines^10^ against cell doubling time, which revealed a significant correlation, consistent with the interpretation that Module 10 is functionally associated with the cell cycle (Fig. 6h, Pearson *R* = −0.428). All together, these analyses indicate that our data-driven approach to module detection can identify coordinately regulated molecular features associated with quantitative phenotypic responses and that these findings generalize to independent data sets.

#### Examination of module activity to elucidate the molecular basis of ligand-induced phenotypic responses

**Fig. 7:**
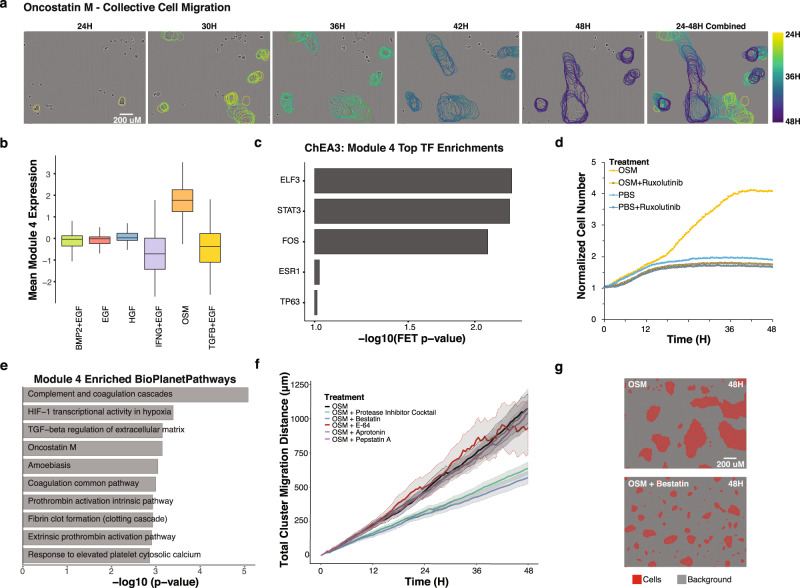
Analysis of molecular modules identifies functional relationships between molecular and phenotypic responses to OSM. **a** OSM induces the formation of cell clusters that undergo collective migration and merge to form large clusters. Representative tracks of OSM-induced cluster migration are shown from 24H to 48H after OSM treatment. Cluster outlines are colored by experimental time point. All images are set to the same scale. **b** Boxplot shows the mean expression of molecular features in Module 4 for each of the six ligand treatments. The boxplots’ lower and upper hinges correspond to the first and third quartiles. The median is shown as the center line. The upper whisker extends from the hinge to the largest value no further than 1.5 * IQR from the hinge (where IQR is the inter-quartile range, or distance between the first and third quartiles). The lower whisker extends from the hinge to the smallest value at most 1.5 * IQR of the hinge. Data in Supplementary Data 15. **c** Barplot showing the top 5 enriched transcription factors inferred for the Module 2 genes in Chea3. Data in Supplementary Data 17. **d** The JAK/STAT inhibitor Ruxolitinib inhibits cell growth in the presence of OSM. Line graph shows the relative number of cells across time. PBS (phosphate buffered saline) treatment serves as a control. **e** Barplot of the top 10 enriched pathways in Bioplanet using the module 4 RNAseq gene set. Data in Supplementary Data 20. **f** OSM-induced collective migration is mediated by protease activity. Line graph shows the accumulated cluster migration distance after OSM + /− a protease inhibitor cocktail and its individual components including bestatin, E-64, aprotonin, and pepstatin A. Solid lines show the population average and gray shaded regions indicate 95% confidence intervals of the mean distance travelled at each time point. **g** False color phase contrast images at 48H show that bestatin inhibits the formation of large cell clusters when given in conjunction with OSM. Cells are colored red and the background is colored gray.

In our final analysis, we illustrate how the modules can be examined to provide insights into the molecular basis of complex phenotypic responses. Here, we focused on OSM, a member of the IL6 cytokine family implicated in immune function, developmental processes, and tissue remodeling^88^. OSM stimulated proliferation and was the only ligand in our panel that induced collective migration, a complex phenotype in which individual cells form tight clusters that undergo migration (Fig. 7a, Supplementary Movies). To date, the molecular correlates of collective cell migration are not well understood, and our dataset provides a unique opportunity to study this behavior.

To gain insight into the molecular features underlying this unique phenotype, we focused on modules that were strongly induced by OSM, including Modules 4, 12 and 13 (Supplementary Fig. 7). Features in Module 4 were of particular interest, as this module was selectively induced by OSM (Fig. 7b and Supplementary Data 15). Module 4 includes RPPA features pSTAT3, P-Cadherin, Connexin-43, and Hif-1-alpha as well as top-ranked transcription factors *ELF3, STAT3, TP63*, and *FOS* from ChEA3 analysis (Fig. 7c and Supplementary Data 17). P-Cadherin and Connexin-43 are intriguing, as they are implicated in the cell adhesion contacts required for mediating the observed clustering phenotype^89,90^. Based on the coordinated changes in STAT3 across modalities, we tested the functional importance of this axis with Ruxolitinib, a JAK/STAT inhibitor. We found that addition of Ruxolitinib in the presence of OSM strongly inhibited both the growth of cells and cell migration, confirming the importance of JAK/STAT signaling in mediating responses to OSM (Fig. 7d and Supplementary Movies 8 and 9).

To probe more deeply into the Module 4 RNAseq features and augment our Reactome enrichment findings, we tested for enriched pathways using BioPlanet^91^ (Fig. 7e and Supplementary Data 20). One of the top pathway hits in this analysis was ‘OSM’, which serves as a validation of the module approach. The most enriched pathway was ‘complement and coagulation cascades’, two linked processes driven by a series of proteases to stimulate innate immunity and blood clotting^92^. This suggested that protease activity may be critical for mediating OSM-induced cluster migration. To examine the role that proteases play in cluster migration, we treated MCF10A cells with OSM in the presence of a cocktail of five protease inhibitors and found reduced cluster migration, indicating the importance of protease activity in mediating this phenotype (Fig. 7f). We next tested individual components of the protease cocktail and found limited effects of aprotinin, E-64, and pepstatin A. However, with bestatin, an aminopeptidase inhibitor, we observed formation of cell clusters but a failure of these clusters to migrate and merge (Fig. 7g). These functional studies developed from the module analysis implicate aminopeptidase activity as a critical mediator of OSM-induced collective cell motility in MCF10A cells. Overall, our approach to leverage responses to multiple perturbations enabled identification of molecular programs associated with complex phenotypic responses including cluster migration and cell proliferation.

## Discussion

We leveraged the LINCS Consortium framework to systematically quantify the phenotypic and molecular responses of MCF10A mammary epithelial cells after treatment with a diverse panel of ligands. Analysis of this dataset revealed robust molecular and phenotypic responses and enabled identification of ligand-specific signatures, integrated molecular modules, and linkage of phenotypic and molecular responses. These data support the idea that deeply examining a single model system subjected to a range of perturbations with measurements across multiple modalities is crucial to understanding complex biological phenomena.

The robust, multimodal dataset enabled a range of computational analyses. For instance, the coordinated use of a diverse panel of molecular assays facilitated comparisons of the information carried by each assay and revealed that RNAseq and ATACseq assays had the greatest ligand-associated signal. Differences in information content between assays may be due to: intrinsic differences in molecular modalities, the signal available in a particular assay, or differences in the number and diversity of biologically meaningful features in each assay. These findings suggest that comprehensive assays such as RNAseq are well-suited for discovery-based screens or experiments that examine large panels of perturbagens, whereas targeted assays such as CyCIF—which can be adapted through inclusion of different biomarkers—would be expected to excel in more focused hypothesis-driven studies^49,50^.

In our integrated analysis, we joined epigenomic, transcriptional and proteomic changes into co-regulated modules. Critical for this analysis was the use of ligands that stimulate diverse and partially overlapping pathways, as this enabled identification of molecular features that were subtly and variably induced by multiple ligands. We analyzed the modules to identify linkages between molecular features and phenotypic responses. For instance, we identified a set of co-regulated molecular features strongly associated with cell cycle, including both canonical transcriptional factors, pathways, and proteins as well as features that have been implicated but not confirmed in cell cycle regulation, such as PASK^84,85^. Importantly, we showed that this cell cycle module, which was derived from integrating all 6 ligand perturbations, could generalize to independent datasets comprised of multiple cell lines. Some modules were semi-correlated and contained similar biological programs, as indicated by enrichment of shared pathways and TF programs. Alternate methods to identify modules that permit partial membership of individual features may allow a more nuanced identification of the relationship between molecular features and phenotypic responses^93^.

Our findings support the idea that systematically testing multiple perturbations of a single model system can identify molecular programs that are operable in distinct cellular contexts. We assert that identification of these generalizable programs was possible precisely because we used multiple perturbations in a single model system. However, there are also limitations to this approach. For example, a molecular or phenotypic response to a perturbation could be context dependent and may not be observed in other cell lines or model systems. Further exploration of additional cell lines using a panel of perturbations could facilitate identification of the context dependence of the responses we observed and also would enable refinement of the underlying regulatory networks. Indeed, in the disease setting, the assessment of molecular and functional responses in panels of cell lines has proven a powerful approach to identify biological mechanisms common to different disease states^6,8–11,13,20–22^. Additionally, an expanded set of perturbations, including ligands, small molecule inhibitors or siRNAs that target other signaling pathways could help to refine the modules we identified here and could also lead to identification of additional functional modules and molecular networks.

Our live-cell imaging studies revealed the induction of phenotypic responses in response to ligand perturbation. OSM uniquely induced MCF10A cells to form tight cell clusters that underwent collective migration. We used our module analysis to explore the molecular basis of this complex phenotypic response and examined modules that were uniquely induced by OSM. Experimental validation identified functional links between OSM-induced molecular and phenotypic responses: protease activity was required for collective cell migration while STAT activation was required for proliferation. Our findings add to the substantial literature that implicates proteases in modulating interactions between cellular and extracellular signals^94^. Future studies that examine the role of other Module 4 features will be needed for a complete understanding of the molecular basis of OSM-induced collective migration. For example, additional complex phenotypic responses could be investigated by growing MCF10A cells as 3D organoids^45^.

Together, our findings indicate that this LINCS ME perturbation dataset will serve as a robust and valuable resource for community-wide analysis and exploration. This resource can be utilized by the broader community to gain deeper insights into biological processes such as the molecular basis of different phenotypes, the molecular and phenotypic impact of particular ligands, and how specific molecular features are modulated by perturbation. Additionally, these data can serve as a resource for computational scientists to examine relationships between different molecular modalities, to develop methods for identifying molecular networks, or to elucidate the temporal relationships between different types of molecular changes. We also envision expansion of the dataset to include additional molecular measurements (e.g. single-cell RNAseq, single-cell ATACseq, and single-cell proteomics) and perturbation with different ligand combinations. Finally, while MCF10A represents a robust model of epithelial cell biology, analysis of the phenotypic and molecular responses observed in other cell models will be important for establishing broad generalizability of different findings. Our study provides a blueprint of the considerations for generating large-scale, high-quality multi-omic perturbation data, and serves as a reference set against which other cell types could be compared. In addition, our results could be used to help guide future studies by informing the optimal assay, perturbation or time point for more hypothesis-driven studies.

## Methods

### General considerations

The technical reproducibility of a data resource such as the one we described here is paramount. To support the development of a robust resource, we carefully planned all experiments to minimize technical artifacts and batch effects. Some aspects of the design of this data generation exercise were piloted in an earlier LINCS-wide study of reproducibility that we published jointly with co-authors of this manuscript (Niepel, et al. *Cell Systems* 2019^51^). Specifically, in this study, we considered the following, which are described in more detail in the subsequent sections: (1) Cell line evolution (drift): Whenever possible, cell culture was performed at OHSU to minimize technical variation. Given the nature of the CyCIF assay, it was necessary for HMS to perform cell culture at their site. To control for cell line evolution, several cell aliquots were frozen down at OHSU prior to the start of the experiment. These aliquots were shared with HMS for CyCIF data generation. For each sample collection (described below), we used a fresh aliquot of cells and ensured that cells were minimally passaged during sample generation. (2) Reagent batch-to-batch variation: To minimize variation due to reagents, common stocks of media and ligands were used for all sample generation at OHSU and HMS. (3) Cell culture protocols: OHSU and HMS used common cell culture protocols to minimize technical and biological differences. (4) Experimental collections: The large number of cells required for each assay necessitated that we split the gathering of samples into different collections to ensure feasibility of cell culture, treatment, and harvest. Each collection had at least three biological replicates that took approximately a month to generate. Details about which assays were included in each collection are shown in Fig. 1e. To test for consistency across collections, we performed functional analyses on each of the biological replicates and found that they were broadly similar. Results of comparison from collections 1 and collections 2 are shown in Fig. 2 and indicate concordant responses.

### Methodological rationale

A comprehensive study of how cells modulate their cellular and phenotypic responses to extracellular signals is critically important for understanding a variety of biological processes including cell state control, development, and diseases such as cancer. This includes identification of the molecular networks that are directly modulated, the duration and extent of modulation, how one perturbation compares to another, and identification of feedback mechanisms. Additionally, identification of the molecular networks that underlie phenotypic responses such as cell migration or proliferation remains challenging; for example, a TGFB network is not synonymous with a proliferation network despite TGFB treatment modulating proliferation.

These questions on ligand and phenotype networks have been difficult to address because they require identifying a sufficient range of perturbations that modify multiple phenotypes in a single cell type, and then using experimental and quantitative approaches that can isolate the underlying networks from secondary responses (feedback) and multiple complex phenotypic responses (e.g., migration and proliferation are both stimulated by EGF). Furthermore, these experiments are difficult to conduct across cells lines because a ligand perturbation in one cell type may not be equivalent to a ligand perturbation in a second cell type due to differences in the receptors that are expressed, the abundance of those receptors, downstream signaling components, transcription factors, and the underlying state of the cell. In addition, without a large reference dataset it remains unclear the number and type of perturbations to analyze, the optimal time points to collect, the type of assays to measure, and what bioinformatic tools are necessary to integrate all this information identify these networks. An additional challenge is that it is inherently difficult to generate comprehensive multi-omic data as it requires expertise in the collection and analysis of each individual data type as well as development of methods to integrate data types together.

Motivated by this, we leveraged the LINCS consortium, comprised of multiple laboratories with diverse expertise, to create a comprehensive dataset on a single cell type that would be of broad use to the research community to mine for biological insights, develop novel computational analyses, and to serve as a guide of considerations for building multi-omic perturbation data sets. To maximize the richness of the resultant data resource, we decided to test multiple perturbations in a single cell line, which provides several advantages over testing fewer perturbations in multiple cell lines. First, this increases experimental tractability as perturbagens and assay growth conditions need only be optimized for a single sample, and second, the starting state of cells is the same in all samples, which enables a range of responses to be compared and leveraged against each other to isolate individual networks associated with different phenotypic responses. One disadvantage of using a single cell line is that it is not possible to directly address what portion of a perturbation response is cell type-specific compared to the portion that is conserved across multiple cell types. Balancing these considerations with available resources, we chose to use a single cell type for this study. Our approach enabled isolation of primary from secondary response; for example, we were able to identify molecular changes specific to EGF and separate these from changes associated with secondary effects such as proliferation. This type of approach has been deployed for deep analysis of other model systems, including drosopholia^11^ and c.elegans^12,13^.

### Cell culture methods

To decrease unwanted biological variation and ensure comparable results across data types, MCF10A cells were frozen in a single batch at the MD Anderson Cancer Center and used by both OHSU and HMS from the frozen batch with limited passaging. Cell identity was confirmed by short tandem repeat (STR) profiling and cells tested negative for mycoplasma.

Two media formulations were used in these experiments. For routine growth and passaging cells were cultured in growth media (GM) composed of DMEM/F12 (Invitrogen #11330-032), 5% horse serum (Sigma #H1138), 20 ng/ml EGF (R&D Systems #236-EG), 0.5 µg/ml hydrocortisone (Sigma #H-4001), 100 ng/ml cholera toxin (Sigma #C8052), 10 µg/ml insulin (Sigma #I9278), and 1% Pen/Strep (Invitrogen #15070-063). For perturbation experiments, we used growth factor free media—which we termed experimental media (EM)—that was composed of DMEM/F12, 5% horse serum, 0.5 µg/ml hydrocortisone (Sigma #H-4001), 100 ng/ml cholera toxin (Sigma #C8052), and 1% Pen/Strep (Invitrogen #15070-063). For each experiment, MCF10A cells were grown to 50-80% confluence in GM and detached using 0.05% trypsin-EDTA (Thermo Fisher Scientific 25300-054). Following detachment, 75,000 cells were seeded into collagen-1 (Cultrex #3442-050-01) coated 8-well plates (Thermo Fisher Scientific 267062) in GM. Six hours after seeding, cells were gently washed with PBS and EM was added. Following 18 hours of incubation in EM, cells were treated with ligand in fresh EM media as follows: 10 ng/ml EGF (R&D Systems #236-EG), 40 ng/ml HGF (R&D Systems #294-HG), 10 ng/ml OSM (R&D Systems #8475-OM), 20 ng/ml BMP2 (R&D Systems #355-BM) + 10 ng/ml EGF, 20 ng/ml IFNу (R&D Systems #258-IF) + 10 ng/ml EGF, 10 ng/ml TGFβ (R&D Systems #240-B) + 10 ng/ml EGF. The addition of ligand started the experimental clock. Samples were then collected at 1, 4, 8, 24 or 48H following ligand addition as shown in Fig. 1.

Eight-well plates were coated with 20 µg/cm^2^ collagen-1 in a mixture that mimicked the buffering and structural characteristics of MEMA spots: 200 µg/ml collagen-1 (Cultrex #3442-050-01), 10% v/v glycerol (Sigma G5516), 5 mM EDTA pH 8 (Invitrogen 15575), and 100 mM Tris-HCl pH 7.2 (Sigma T2069) in PBS. Plates were rocked at RT for 1 h. Remaining coating mixture was gently aspirated and plates were washed twice with sterile PBS. Wells were allowed to dry completely by leaving the plate uncovered in a laminar flow hood before being stored in a benchtop desiccator for a minimum of three days and maximum of six months before use.

After identification of the 6 ligand treatments, samples were generated over three collection periods. The first collection was completed at OHSU in the Fall of 2017 when RPPA, RNAseq, ATACseq, L1000, and IF samples were collected. The second collection was completed at OHSU in the Winter of 2018 and included GCP, L1000, and IF samples. The third collection was collected at HMS in the Summer of 2018 and included CyCIF and L1000 samples.

### Microenvironment microarray (MEMA)

We used previously established high-throughput MEMA screens to identify microenvironmental factors that strongly influence growth^14,48^. The key aspects of the MEMA assay are comprised of a set of printed insoluble proteins and a panel of soluble ligands. In brief, a panel of 48 insoluble proteins were printed into 8-well cell culture plates with an Aushon printer, forming 350 um diameter spots on which cells can grow. Each matrix protein was mixed with collagen I to improve printing and cell attachment, and printed in ∼15 replicate pseudo-random locations. 22,000 cells per well were added to replicate arrays and grown in experimental media for 18H. Following this, the media was exchanged and appropriate concentrations of a panel of 63 soluble ligands were added to each well. To account for the influence of EGF on MCF10A proliferation, we tested one set of arrays with 10 ng/ml EGF and the other without added EGF. Arrays were returned to the incubator for 71 hours, after which 1uM EdU was added to the medium for 1 hour. Cells were then fixed in 2% PFA at RT, and stored at 4 °C in PBS. After fixation, cells were permeabilized with 0.3% Triton X-100 for 25 minutes at RT. Array-bound cell staining was performed with KRT14 (Abcam, 1:200), CellMask, and DAPI (ThermoFisher, 1:10,000).

Arrays were imaged on a customized automated high content fluorescence microscope platform (Nikon HCA) and resultant image data was output to an OMERO image database^95^. Cells were segmented and intensity levels were calculated using CellProfiler^96^. The resulting MEMA data was preprocessed and normalized using open-source R software available from (https://github.com/MEP-LINCS/MEP_Processing). The spot cell count was based on the DAPI stained nuclei. EdU intensity was auto-gated to label cells as EdU+ and the proportion of EdU+ cells in each spot was reported to measure proliferation. Each intensity and morphology signal was independently RUV normalized in a series of matrices with arrays as the rows and spots as the columns^97^. The RUV controls were the residuals created by subtracting the replicate median from each spot value. After RUV normalization, bivariate LOESS normalization was applied to the normalized residuals using the array row and array column as the independent variables. After normalization, the ∼15 replicates of each condition were median summarized to the MEP level.

### MCF10A dose optimization

We used a three-step process to identify ligands and optimize doses for this large-scale perturbation experiment. Importantly, rather than use the same dose concentration for each ligand, we ran pilot studies to identify functionally relevant concentrations. First, we used a high-throughput MEMA screen to identify ligands that modulated proliferation. Second, we prioritized hits from the MEMA screen by selecting a panel of ligands that target diverse receptor classes (cytokine, growth factor, TGFB family) and which targeted highly expressed receptors. Third, for each of the 6 candidate ligands, we performed dose-response studies to identify the relationship between ligand dose and change in cell numbers after perturbation. MCF10A cells were plated on collagen coated 24-well plates in full growth media for 7 hours at which point the media was exchanged for experimental media. Following 18 hours in experimental media, fresh experimental media was added with 7 doses of OSM, EGF, and HGF individually, or with seven doses of BMP2, IFNG, and TGFB in combination with 10 ng/ml EGF. After 72 hours in ligand containing media, cells were fixed, stained with DAPI, and imaged on the ScanR microscope. Cell counts from the images were quantified using Cell Profiler and normalized based on the number of cells present in the 10 ng/ml EGF condition. These dose-response experiments were performed in biological triplicate. From the resultant curves, we chose supramaximal doses for each ligand treatment, reasoning that this would ensure robust changes in cell number and minimize effects due to ligand depletion over the course of the 48H assay.

### OSM validation experiments

To assess responses to JAK/STAT inhibition MCF10A cells were plated in 24-well collagen coated plates. Following the media changes, cells were treated with 10 ng/ml OSM, 10 µM ruxolitinib (Selleck Chemicals #S1378) and Nuclight Rapid Red Dye (Essen Bioscience #4717) to label nuclei and count cells across time. Cells were then placed in the IncuCyte S3 and imaged every 30 minutes for 48 hours using phase contrast and red fluorescent filter sets. Cell number was quantified in Cell Profiler by counting the number of fluorescent nuclei in each frame and normalizing counts to time 0H.

To assess cell responses to protease inhibitors cells were plated in 24-well collagen coated plates, underwent the standard media changes and then at time 0H treated with 10 ng/ml OSM and either a protease inhibitor cocktail at a 1:400 dilution (Sigma-Aldrich #P1860), 40 µM bestatin (Sigma-Aldrich # B8385), 800 nM aprotinin (Sigma-Aldrich # A1153), 10 µM E-64 (Sigma-Aldrich # 324890), 1.45 µM pepstatin (Sigma-Aldrich # P5318). Cells were then placed in the IncuCyte S3 and imaged every 30 minutes for 48 hours.

Phase contrast images were registered using a custom ImageJ script and then imported into the Baxter Algorithms cell tracking software^98^. Clusters of cells with an area greater then 1000 pixels (~5 cells) were tracked using default parameters. Cell cluster tracks were then analyzed to quantify migration. Speed, displacement, mean squared displacement, and the cumulative distance traveled was calculated for cell clusters.

### Live-cell imaging

Well plates were placed in the IncuCyte FLR and phase contrast images were acquired every 30 minutes for 48 hours. Individual cells were manually tracked using the Fiji^99^ plugin MtrackJ^100^. Custom R scripts were used to quantify the migratory behavior of individual cell lineages. In brief, starting at the last time slot of each lineage, one cell was randomly selected and traced back through mitotic events until T0. Migration distance for each lineage was then calculated as the sum of the distances in pixels along the path between each image. To compare migratory behavior across different ligand treatments, we performed an ANOVA followed by Tukey’s Honestly Significant Difference test in R. Ligand treatments with p-value < 0.05 were deemed significantly different.

### Immunofluorescence

Prior to fixation, cells were pulsed with 10 µM EdU (Thermo Fisher Scientific C10357) for 1 hour under standard culture conditions. Cells were then fixed for 15 minutes with 2% paraformaldehyde (Electron Microscopy Sciences #15710) and permeabilized for 15 minutes with 0.01% Triton X-100 in PBS. Cells were then stained with CellMask (Thermo Fisher Scientific #H32713) for 30 minutes at RT, followed by fluorescent labeling of incorporated EdU for 1 hour at RT (Thermo Fisher Scientific C10357). Finally, cells were stained with a keratin 5 polyclonal antibody (BioLegend #905501) at 1:800 overnight at 4 °C, followed by an anti-rabbit 488 secondary antibody (Thermo Fisher Scientific A21206) at 1:300 and Dapi (PromoKine PD-CA707-40043) at 0.5 µg/µL for 1 hour at RT.

Fixed cells were imaged on an Olympus ScanR microscope. DAPI channel images were imported into Ilastik for pixel classification^101^. A set of 20 images per plate were randomly selected and used for training. Pixels were classified as either nuclei or background using all default intensity, edge, and texture features, and with smoothing filters ranging from 0.3 – 10 pixels. Probability maps were then exported from Ilastik into CellProfiler version 3.1.8 for object segmentation^102^. Nuclei were identified using the global Otsu method with a threshold smoothing scale of 1.35. Clumped nuclei were separated based on intensity with a smoothing filter of 12 pixels. Cytoplasm compartments were assigned to nuclei by a 10-pixel donut expansion from each nucleus. Cytoplasm and nuclear Intensity, size, and morphology data was then exported into RStudio (RStudio Team, 2015). The values are analyzed as populations that have been median summarized from the cell-level data to the image or field level. The field level data are then median summarized to the well level. The EGF time course normalized values are the raw values divided by the corresponding EGF value at the same time point within the same replicate set. The preprocessing and QA script is at https://github.com/MEP-LINCS/MDD/tree/master. All samples passed qualitative QC inspection that the integrated DAPI intensity has the expected bimodal distribution.

### Phenotype analysis

All phenotypic quantifications were derived from immunofluorescent cell-level data. Cell cycle phase was determined by analysis DAPI intensity: each cell was classified into either G1 or G2M cell cycle phase by clustering cells into two groups based on total nuclear DAPI intensity. The Forgy k-means algorithm was used for clustering (R stats package), with the number of centers set to two. DAPI thresholds for classification were manually inspected, and multinucleated and poorly segmented cells were removed from further cell cycle analysis. KRT5 intensity was calculated as the mean intensity value of KRT5 in the cytoplasmic cell compartment.

Three spatial metrics were computed to quantify treatment induced changes in cell clustering and dispersal. The number of neighbors for each cell was calculated by quantifying the number of cell centroids within 100 pixels of a cell’s centroid. Cells with coordinates less than 100 pixels from the image border were excluded. Nearest neighbor distances were determined by measuring the pixel Euclidean distances of each cell centroid to the centroids of the four nearest cells in the imaging field. To account for variations in image cell count, the mean nearest neighbor distances for each image were normalized by the expected mean distance to the nearest neighboring cell if the cells were distributed randomly^103^. The number of cells per cluster was computed in a two-step process: first performing mean shift clustering on the cell centroid coordinates for each image, using the R package LPCM (v 0.47), and then computing the mean number of cells per cluster.

To compare phenotypic responses across treatments, we analyzed quantifications of the immunofluorescent images 48 hours after treatment. The Kruskal-Wallis test was used to test for overall treatment dependent differences. Pairwise comparisons between treatments were then conducted using Pairwise Wilcoxon Rank Sum Tests followed by an FDR multiple comparisons correction. A stringent significance threshold of q-value < 0.05 was used to aid in identification of the most differentially induced phenotype features.

### Reverse phase protein array sample preparation

Cells were washed twice with ice-cold PBS followed by collection by manual scraping in 50-100 µL of lysis buffer (1% Triton X-100, 50 mM HEPES pH 7.4, 150 mM NaCL, 1.5 mM MgCL_2_, 1 mM EGTA, 100 mM Na pyrophosphate, 1 mM Na_3_VO_4_, 10% glycerol, 1x cOmplete EDTA-free protease inhibitor cocktail (Roche #11873580001), 1x PhosSTOP phosphatase inhibitor cocktail (Roche #4906837001)). Lysates were incubated on ice for 20 minutes with gentle agitation every 5 minutes and then centrifuged at 14,000 rpm for 10 minutes at 4 °C. Supernatant was collected into a fresh tube, quantitated by BCA assay, and the appropriate volume was combined with 4X SDS sample buffer (40% glycerol, 8% SDS, 0.25 M Tris-HCl, 10% β-Me, pH 6.8), boiled for 5 minutes, and stored at −80 °C. Three sets of replicates were collected over three weeks and submitted to MD Anderson Cancer Center for RPPA testing.

### Reverse phase protein array preprocessing and QC

Samples underwent standard pre-processing using methods developed at the MD Anderson Cancer Center RPPA core^104^. In brief, the processing steps include the following: 1) Convert raw data from log2 value to linear value. 2) Determine median for each antibody across the sample set. 3) Calculate the median-centered ratio by dividing each raw linear value by the median for each antibody. 4) Assess sample quality by computing a correction factor (CF.1) for protein loading adjustment for each sample as the median of the median-centered ratio values from Step 3 for all antibodies. Samples with correction factors above 2.5 or below 0.25 are considered outliers and discarded. 5) Compute the normalized linear value by dividing the median-centered ratio from Step 3 by CF.1. All samples passed MDACC’s quality checks and are included in the dataset. The normalized RPPA log2 values are joined with their experimental metadata and stored on Synapse as level 3 data. Replicates are median summarized and stored as Level 4 data.

### RNAseq sample preparation and sequencing

Following treatment protocols described, at the appropriate time point wells were aspirated and cells were harvested by scraping in 600 µl of RLT Plus buffer (Qiagen) plus 1% β-ME. Samples were flash frozen in liquid nitrogen and stored at −80 °C prior to RNA extraction. Total RNA was extracted from frozen using a Qiagen RNeasy Mini kit. Columns were DNAse treated following the recommended protocol of the manufacturer.

RNA concentration and purity was determined by UV absorption using a Nanodrop 1000 spectrophotometer. All samples had 260/280 absorption ratios of at least 2.0, indicating successful isolation of RNA from other nucleic acids. RNA integrity was assessed using an Agilent 2100 Bioanalyzer with an RNA 6000 Nano Chip. RNA integrity numbers (RIN) were calculated from Bioanalyzer electropherograms using the “Eukaryotic Total RNA Nano” program of the Bioanalyzer 2100 Expert software (B.02.08.SI648). RIN values were in the 8.5-10 range, indicating high-quality RNA, with one exception (TGFB_48_C1_B; RIN = 6.9). UV absorption measurements and RIN values are available on Synapse (10.7303/syn12550434).

cDNA libraries were prepared from polyA-selected RNA using an Illumina TruSeq Stranded mRNA library preparation kit. 100-bp single-end reads were sequenced on an Illumina HiSeq 2500 Sequencer, with a target of 60 M reads per sample.

### RNAseq pre-processing and QC

Sequence preprocessing and alignment was performed using a Docker-based pipeline^105^. 100-bp single-end reads were trimmed of Illumina adapter sequences using TrimGalore (v. 0.4.3), a wrapper for CutAdapt (v. 1.10) and FastQC (v. 0.11.5). A minimum of 1-bp overlap with the adapter sequence (AGATCGGAAGAGC) was required for trimming. After trimming, reads with a length < 35 bp were discarded. Trimmed reads were aligned to the GENCODE V24 (GRCh38.p5) assembly of the human genome using the Kallisto pseudo-alignment software (v. 0.43.0). Kallisto, using the following parameters: --bias -b 30 --pseudobam.

Gene-level quantifications were produced from transcript-level abundance estimates using the R (v. 3.5.0) package tximport (v. 1.8.0). Mapping between gene/transcript identifiers was done using the biomaRt package (biomaRt v. 2.36.1) with the ENSEMBL_MART_ENSEMBL biomart and the hsapiens_gene_ensembl dataset. Gene-level quantifications were imported to DESeq2 (v. 1.24.0)^106^. The fpkm function of DESeq2 was used to normalize data for library size and gene length differences, and fpkm values were log2 transformed with an added pseudocount of 1.

### Transcription Factor enrichment scores

Single-sample enrichment scores were calculated for 297 transcription factor target gene sets obtained from the CHEA3 ReMap_ChIP-seq^72^ using the R package GSVA (v. 1.32.0)^107^. A minimum expression filter was used for expressed genes; genes were retained only if expressed at a minimum of 0.5 log2(fpkm + 1) in a minimum of 3 samples. Enrichment scores were calculated from filtered RNAseq data, in units of log2(fpkm + 1), using the argument “method = ‘ssGSEA’”.

### Identification of differentially expressed genes

For each ligand treatment, we performed a differential expression analysis on the RNAseq gene-level summaries with the R package DESeq2 (1.24.0), with shrunken log2 fold change estimates calculated using the apeglm method. We used the Benjamini-Hochberg method to correct p-values for multiple comparisons and set a threshold of q-value < 0.01 and shrunken log2 fold change > 1.5 or < −1.5 to indicate significance.

### Pathway enrichment of ligand-induced signatures

We used Gene Set Enrichment Analysis (GSEA) to identify the pathways enriched by each ligand treatment. Specifically, we used Gene Set Enrichment Analysis 4.1.0 downloaded from https://www.gsea-msigdb.org/gsea/index.jsp to assess enrichment of the MSigDB Hallmark Pathways in the Level 3 data. For each 24H ligand treatment sample, we computed log2 fold-change against CTRL_0 from the Level 3 RNAseq data.

### ATACseq sample preparation and sequencing

ATACseq samples were collected following the Omni-ATAC protocol^108^. Briefly, MCF10A cells were washed once with PBS and detached from the plate using trypsin. Cells were then counted using a Countess (Invitrogen), and 50,000 cells per condition were distributed to 1.5 ml centrifuge tubes and spun at 500 RCF for 5 min. The supernatant was removed and the cell pellet was resuspended in 500 µl of PBS and spun again at 500 RCF for 5 min. The supernatant was removed again, and the cell pellet was resuspended in 50 µl of cold ATAC resuspension buffer (RSB) containing 0.1% NP40, 0.1% Tween-20, and 0.01% digitonin by pipetting up and down three times. After 3 min on ice, 1 ml of cold RSB containing 0.1% Tween-20 was added, and the tube was inverted three times to mix. The nuclei were then pelleted by centrifugation at 500 RCF for 10 min at 4 °C. The supernatant was then carefully aspirated, and the nuclei were resuspended in 50 µl of transposition buffer (25 µl 2x TD buffer (Illumina), 2.5 µl transposase (Illumina), 16.5 µl PBS, 0.5 µl 1% digitonin, 0.5 µl 10% Tween-20, and 5 µl H2O). Samples were then placed in a pre-warmed (37 °C) thermomixer and mixed for 30 min at 100 RPM. Transposed fragments were then purified using a Qiagen MinElute column and frozen at −80 °C for further processing.

The remaining steps of the Omni-ATAC protocol were performed by the OHSU Massively Parallel Sequencing Shared Resource. Transposed fragments were preamplified with 5 rounds of PCR. Afterward, 5 µl of the pre-amplified mixture was used for a qPCR reaction to determine the concentration of tagmented DNA. After calculating the concentration of tagmented DNA, pre-amplified samples were diluted with elution buffer to a final concentration of 5 µM. Six samples had an undiluted DNA concentration below 5 µM and were not diluted. 5 µM pre-amplified samples were amplified for 3 additional PCR cycles.

Tagmented DNA was pre-amplified with 5 rounds of PCR (72 °C for 5 min, 98 °C for 30 s, then 5 cycles of [98 °C for 10 s, 63 °C for 30 s, 72 °C for 1 min]). PCR reactions contained 20 µl eluate, 25 µl NEBNext 2x MasterMix, 2.5 µl 25 µM i5 primer and 2.5 µl 25 µM i7 primer. The DNA concentration of the pre-amplified samples was assessed by qPCR. 5 µl of pre-amplified mix was added to 3.76 µl sterile water, 0.5 µl 25 µM i5 primer, 0.5 µl 25 µM i7 primer, 5 µl 2x NEBNext master mix, and 0.24 µl 25x SYBR Gold (in DMSO). Samples were amplified for 20 cycles of [98 °C for 10 s, 63 °C for 30 s, 72 °C for 1 min]. DNA concentration was calculated, and pre-amplified samples were diluted to a final concentration of 5 µM. Six samples had an undiluted DNA concentration below 5 µM and were not diluted. 5 µM pre-amplified samples were amplified for 3 additional PCR cycles. 100 bp PE reads were sequenced on an Illumina HiSeq 2500 Sequencer by the OHSU Massively Parallel Sequencing Shared Resource with a target of 20 M reads per sample.

### ATACseq preprocessing and QC

ATACseq files were processed and aligned using the ATACseq (1 -> 3) workflow on the AnswerALS Galaxy server (answer.csbi.mit.edu). Reads were trimmed of adapter sequences and low-quality bases using Trimmomatic (Galaxy version 0.36.5). Reads were trimmed of low-quality bases (Phred score < 15) at the read start or end, and Nextera adapter sequences (CTGTCTCTTATA) were trimmed from read ends (minimum of a 2-bp overlap required for trimming). Reads were aligned to the human genome (hg38) using Bowtie2 (Galaxy version 2.3.4.1) in paired-end mode with otherwise default settings. BAM files were filtered to remove secondary alignments, unmapped reads, and mitochondrial DNA alignments using ngsutils bam filter (Galaxy version 0.5.9). PCR duplicates were detected and removed using Picard MarkDuplicates (Galaxy version 2.7.1.2). The de-duplicated, filtered BAM file was used for peak calling and quantification. Peaks were called using MACS2 (Galaxy Version 2.1.1.20160309.5) using the following parameters: -format BAMPE -nomodel -extsize 200 -shift −100 q value 0.01.

ATACseq sample quality was assessed by calculating the fraction of reads in peaks (FRiP). Before calculating FRiP, a consensus peakset was generated for all samples by taking the union of all peaks called in all samples and merging any overlapping peaks, using the R (v. 3.6.1) package DiffBind (v. 2.12.0)^109^. For each sample, FRiP was then calculated by counting the proportion of reads in the de-duplicated, filtered BAM file that align within the consensus peakset. A minimum FRiP threshold of 0.15 was applied to remove samples with low levels of chromatin enrichment. Thirteen ATACseq samples did not pass the QC due to low FRiP scores; the fragment length distributions of these samples also lack the periodic peaks caused by nucleosome patterning. These low-quality samples likely are the result of fragment over-transposition due to a high Tn5-transpose-to-cell ratio^110,111^.

### Construction of chromatin accessibility matrix

DiffBind (v. 2.12.0) was used to generate a peak accessibility matrix for the QC-passing samples. First, a consensus peakset was re-generated after removal of low-FRiP samples. The dba.count function was then used to count the number of reads in the de-duplicated, filtered BAM files that overlap with each peak in the consensus peakset. The dba.count argument “score = DBA_SCORE_TMM_READS_EFFECTIVE” was used to output TMM counts normalized to each sample’s effective library size, which is equal to the de-duplicated, filtered library size multiplied by the FRiP. A peak accessibility matrix in units of unnormalized counts was also generated using the dba.count function with the argument “score = DBA_SCORE_READS”.

### Motif enrichment

Transcription factor motif enrichment scores were generated from the TMM-normalized chromatin accessibility data using the R package chromVAR (v. 1.6.0)^112^. ATACseq peaks were annotated with GC content using the addGCBias function of chromVAR and the BSgenome.Hsapiens.UCSC.hg38 genome annotation package. Transcription factor motif position frequency matrices were obtained from the “JASPAR CORE 2018 Homo sapiens” set of motifs^113^. ATACseq peaks were matched to JASPAR motifs using the R package motifmatchr (v. 1.6.0). The expected fraction of reads per ATACseq peak was calculated using the chromVAR function computeExpectations, with the argument “norm = TRUE”. Each sample’s deviation from the expected fraction of peaks in each annotated category was calculated using the function computeDeviations, and deviations were converted to Z-scores using the function deviationScores. Enrichment scores of individual transcription factors were mean summarized to the “family” level as annotated in JASPAR 2018.

### Global chromatin profiling

The GCP assay was performed as previously described in Creech et al.^53^ and Litichievskiy et al.^11^ Cells were washed with ice-cold PBS, then collected by manual scraping in 200 µl of cold PBS. Cells were then pelleted by centrifugation at 1500 RCF at 4 °C for 5 min, resuspended in 1 mL of cold PBS, and spun again as specified. The resultant cell pellets were then flash frozen in liquid nitrogen and stored at −80 °C until further processing. Pellets were thawed and lysed with nucleus buffer, followed by histone extraction by sulfuric acid and precipitation using trichloroacetic acid. Sample input was normalized to 10 µg of histone in H_2_O before being propionylated, desalted (Oasis HLB 5 mg Plate) and digested by Promega trypsin overnight. A second round of propionylation, followed by desalting using C18 Sep-Pak cartridges (Waters) was employed after digestion. Propionylations and digestion were done in an automated fashion on an LT-Bravos system (Agilent). Isotopically labeled synthetic peptides from histones H3 and H4 were added as a reference to each sample prior to MS analysis. Peptides were separated on a C18 column (EASY-nLC 1000, Thermo Scientific) and analyzed by MS in a PRM mode (Q Exactive^TM^-plus, Thermo Scientific)^53^. Detailed protocols of sample preparation steps can be found in https://panoramaweb.org/labkey/wiki/LINCS/Overview%20Information/page.view?name=sops. GCP data was merged with the experimental metadata and stored as level 3 data on Synapse. Replicates were median summarized and stored as level 4 data.

### L1000 sample preparation

L1000 samples were collected as part of three collections. The first L1000 sample collection was generated in parallel to the ATACseq samples. MCF10A cells were washed once with PBS and detached from the plate using trypsin. Cells were then counted using a Countess (Invitrogen) and 50,000 cells per condition were distributed to 1.5 ml centrifuge tubes and spun at 500 RCF for 5 min. The supernatant was removed, and the cell pellet was resuspended in TCL buffer (Qiagen) containing 1% β-Me. For the second and third collections, cells were washed with PBS followed by the addition of TCL buffer (Qiagen) containing 1% β-Me. The cell and buffer mixture was allowed to sit for 30 min and then frozen at −80 °C for further processing. Samples from the first and second sample collections were frozen in 1.5 ml tubes. Samples from the third data collection were frozen in their original 96-well plates. In total there were eighteen plates from the third HMS collection, which contained 21 samples per plate, and there were 190 samples from the first two OHSU collections. All samples were shipped to the BROAD for simultaneous processing on the L1000 platform. The source plates containing original samples were re-arrayed into six 96-well master plates. These master plates contained 21 samples from each of three original source plates, and 32 samples plated directly from tubes. In each of the six master plates, well A1 was left empty to accommodate for internal technical control spike-ins. The six 96-well master plates were then re-arrayed into the final 384 well v-bottom PCR Plates (Eppendorf #951020702).

### L1000 Ligation Mediated Amplification

For L1000 Ligation Mediated Amplification^20^ crude cell lysates were transferred from source plates to 384 well v-bottom PCR Plates (Eppendorf #951020702) assay plates. Oligo dT coated magnetic particles (GE Healthcare #38152103010150) were added to capture mRNA. Plates were then incubated at room temperature on shaker tables for 10 min. The beads were then spun down onto flat magnets and unbound lysate was evacuated by centrifuging upside down on magnet to 800RPM for 30 s. 15 µl of reverse transcription master mix containing SuperScript IV reverse transcriptase was added to the plates and the plates were incubated at 55 °C for 10 min. Plates were again spun down, beads were pelleted on a flat magnet, and the remaining master mix was spun out. Probes were annealed to the first-strand cDNA by addition of 15 µl of Probe Bind master mix, containing 100 fmole of each probe and Taq ligase buffer. Samples were denatured at 95 °C for 5 min, then transferred to a ramping water bath that decreased temperature from 70 °C to 40 °C over six hours. The following day, beads were again spun down on a flat magnet and master mix was evacuated. To ligate probe pairs, 15 µL of Ligation Master Mix was added, containing Taq DNA ligase and ligase buffer. Plates were sealed and incubated at 45 °C for 60 min. Plates were spun down on magnets and ligation master mix was evacuated as with previous steps. 15 µl PCR master mix containing 0.5 mmole of each primer (T3 and 50-biotinylated T7 universal primers), dNTPs, and PlatinumTaq polymerase in reaction buffer was added to each well, and plates were subjected to 29 cycle PCR. This process yielded biotinylated gene and bead (barcode) specific amplicons.

Each barcode corresponds to a complementary sequence on a Luminex bead, allowing the PCR product to be hybridized to a mixture containing per well ~100 each of 500 Luminex analyte colors. The plate was then denatured at 95 °C for 5 min and incubated at 45 °C for 18 h. Beads were pelleted and stained with streptavidin R-phycoerythrin conjugate for ten minutes. Finally, plates were read on Luminex FlexMap 3D Flow cytometers that detected analyte color (transcript identity) and fluorescence intensity (transcript abundance) for all analytes detected in all wells.

### L1000 preprocessing

To account for differences across the various cell collections, we adapted our standard data processing pipeline in several ways. L1000 data typically use a population-based normalization scheme, known as plate control, as described in Subramanian et al^20^. Here, the EGF treated wells served as the vehicle when conducting vehicle normalization. The standard data processing pipeline was followed, except for the changes at Level 1 and Level 4, described below. L1000 utilizes 10 sets of invariant genes, similar to ‘housekeeping’ genes, to assess quality and in later normalization steps. These gene sets, each containing 8 genes, represent control values that span the spectrum of gene expression, and are ordered according to their overall level of expression, the first level corresponding to the lowest expressing genes, and the 10th corresponding to the highest expressors.

Plates were computationally split at Level 1 (LXB) into subpopulations of wells, each containing only samples from a given time-point and collection combination. The fluorescence intensity values associated with each bead color were subjected to the peak deconvolution step, which separates the two genes associated with each bead color (Level 2). Data were then normalized via L1000 invariant set scaling (LISS), which scales the expression levels of the 978 measured landmarks in each well to the 80 control genes in the invariant gene set (Level 3). Next, we calculated differential expression using EGF as the vehicle control. Robust z-scoring was used to calculate differential expression values for each gene, where gene x is compared only to the vector of normalized gene expression of gene x across all EGF samples in that collection/time-point population (Level 4). Finally, individual biological and technical replicates were collapsed into a consensus signature by computing a pairwise Spearman correlation matrix between each replicate signature. The weights for each replicate were calculated by the sum of their correlations to the remaining replicates, summing to 1. The consensus signatures were generated by the linear combination of the replicate signatures using each signature’s weight as the coefficient (Level 5).

### L1000 QC

We used several approaches to assess data quality. First, to assess the quality in each detection plate, we visually inspected and measured the slope of the invariant gene calibration curve for each sample; outliers were omitted. Second, to assess plate effects, we plotted median fluorescence intensity and interquartile range of invariant set 10 across the entire plate. This allowed identification of failed (low signal) wells, tissue culture related plate effects, or wells with abnormally wide ranges in expression across each gene set. Third, to assess the efficacy of the deconvolution algorithm, we determined the number of well/analyte combinations where two peaks were clearly discernible.

In addition, we computed a transcriptional activity score (TAS) as a composite measure of L1000 transcriptional response. Here signature strength (SS) was computed as the number of genes with a z-score greater than or equal to 2 for each sample, and replicate correlation (CC) was computed as the 7th quantile of the spearman correlation between all pairwise combinations of replicates. TAS is calculated as the geometric mean of SS and CC for a signature, and scaled by the square root of the number of landmark genes, yielding a final score between 1 and 0. QC metrics are available on Synapse (10.7303/syn19416843.1). 2 L1000 samples (1 from C1 and 1 from C3) failed these QC metrics and were removed. Finally, within each sample collection (C1, C2, and C3), we clustered samples based on the Euclidian distances between expression of the 978 measured landmark genes in the Level 3 data, using the R function hclust. Each collection had a small number of outlier samples that showed markedly aberrant expression of the 978 landmark genes and clustered apart from all other samples, in a pattern that was not explained by sample treatment; these 17 samples (3 from C1, 1 from C2, and 13 from C3) were removed. Additionally, 25 samples from Collection 2 lacked an appropriate EGF-treated control on the same 384-well plate and therefore were omitted from the final dataset. In total, 44 L1000 samples (4 from C1, 26 from C2, 14 from C3) were removed from the dataset.

### Cyclic immunofluorescence (CyCIF) sample preparation and imaging

MCF10A cells were seeded 4000 cells/well in 200 µl of GM in collagen coated (as described above) 96 well plates (NUNC, 165305) in technical (multiple wells on the same plate) and biological (experiments separated by a minimum of one cell passage) triplicates. Eight hours after seeding, the cells were washed once with PBS using an EL405x plate washer (BioTek), and 200 µl of EM was added per well. Following an additional 16 hours (24 hours after initial plating), one plate was fixed (time = 0 hours) and EM was aspirated from all wells in the remaining plates using the plate washer and replaced with 200 µl of the appropriate ligand or control treatment.

The treated plates were fixed following incubations of 1, 4, 8, 24, and 48 hours. Cells were fixed in 4% formaldehyde for one hour at room temperature and washed with PBS. Plates were sealed and stored at 4 °C until all replicates were collected. Next, cells were permeabilized with ice cold methanol for ten minutes, blocked in Odyssey buffer (LI-COR) for one hour, pre-stained with secondary antibodies, bleached, and imaged to register background intensities prior to beginning CyCIF^49,50^. For each cycle, cells were stained with three conjugated antibodies, unless otherwise specified, and Hoechst 33342 overnight at 4 °C, washed with PBS, and imaged with an IN Cell Analyzer 6000 (nine fields of view per well, 20x/0.45NA air objective, 2×2 binning) (GE Healthcare Life Sciences). Following image acquisition, fluorophores were chemically inactivated as described^49,50^, and cells then entered the next staining cycle. Refer to Supplementary Data 21 for antibody metadata.

### CyCIF preprocessing and image analysis

A flat field correction profile, generated from all fields on one plate using the BaSiC ImageJ plugin^114^, was normalized to a mean value of one and each image was then divided by it. Image registration was performed with a custom ImageJ script. Segmentation of the nuclei (based on Hoechst staining), and cytoplasm (based on β-catenin staining) was performed with a custom MATLAB (MathWorks) script. Each cell was then divided into four subcellular masks: nucleus, peri-nuclear ring, cytoplasm, and cell membrane for feature extraction, a fifth region including all the cytoplasm (peri-nuclear ring, cytoplasm, and cell membrane together) was also defined. Segmentation was performed on the images acquired in cycle 4 only; the masks were then overlaid on all other cycles for feature extraction. Intensity, texture, and morphology features were extracted for each mask, as appropriate (see Supplementary Data 22 for feature definitions).

### CyCIF QC

Quality control was performed in two steps. In the first step, cells that were washed away over the course of the experiment and those near the edges of the imaging fields that were incompletely captured cycle to cycle due to microscope stage drift were identified and excluded from subsequent analyses. These cells were identified by their high variation in nuclear Hoechst signal between successive cycles (https://github.com/yunguan-wang/cycif_analysis_suite/blob/MCF10A/notebooks/Section2.1_Intensity%20based%20QC.ipynb). If more than 90% of the cells in a field of view failed this QC step, the entire field was removed. The median fraction of lost cells was ~15 % for fields 1-8 whereas an average of 60% of cells were lost from field 9, with a significant number of instances where the fraction of lost cells exceeded 90%. Field 9 was therefore excluded entirely from subsequent analyses. Additionally, for unknown reasons, most of the wells occupying row E on plate 18 exhibited cell loss in excess of 90% leading to the exclusion of all data from those wells in downstream analyses. In the second quality control step, cells with failed cytoplasm segmentation as identified by multinucleation were removed. Multi-nucleated cells were identified by re-segmenting each mask using the Python implementation of Opencv (https://github.com/skvark/opencv-python) and counting the nuclei; cells with two or more nuclei were excluded from downstream analyses (https://github.com/yunguan-wang/cycif_analysis_suite/blob/MCF10A/notebooks/Section2.2_image_based_qc.ipynb). Although masks with two nuclei can represent failed segmentation or truly binucleated cells, visual inspection led us to conclude that these cases were primarily segmentation errors and were therefore excluded from downstream analyses.

### Measuring association between variance and covariates

We applied the Measuring Association between VaRIance and Covariates method to systematically assess the fractional variance explained by each experimental covariate of ligand, time, and replicate^56,57^. Briefly, each dataset was normalized by winsorization at 99% to remove extreme outliers and then median centering within replicate. Next, we performed principal component analysis to reduce the dimensionality of each data set while preserving the variability. A subspace of principal components (PCs) significantly associated with each covariate (ligand, time, replicate) was determined by lasso regression for continuous covariates and silhouette coefficient for categorical covariates. We then quantified the total variance explained by each covariate by summing the weighted variances of all principal components (PCs). Low variance PCs with an eigenvalue of less than 0.7 were unlikely to significantly correlate to any covariates and these discarded PCs were not included in the analysis.

### L1000 drug signature comparison

To compare our results to existing L1000 transcriptional drug signatures^20^ we used the L1000 FWD tool^115^ available at https://maayanlab.cloud/L1000FWD/. We used as input the top 200 most significantly up-regulated and top 200 most significantly down-regulated genes at 24 H relative to CTRL_0. We considered drug signatures with Fisher exact test q-values < 0.2 to be significantly correlated or anti-correlated with our ligand signatures. Finally, we summarized the number of drugs with similar mechanisms of action to identify common patterns.

### Comparison of RNAseq and RPPA assays

To examine the relationship between gene expression and protein abundance, we compared Z-scores calculated from our Level 3 RNAseq and RPPA data for the 222 genes/proteins measured by both assays. We also characterized the relationship between these assays by examining the concordance of genes and protein identified as differentially expressed (compared to time 0) after ligand treatment. Genes meeting an absolute fold-change threshold of 1.5 and an FDR-adjusted q-value of 0.01 were considered differentially expressed (as described in RNAseq methods). RPPA antibodies meeting an absolute log fold-change threshold of 0.5 and an FDR-adjusted q-value of 0.01 were considered differentially expressed. For this analysis, we used a more stringent alpha of q = 0.01 (rather than q = 0.2 used elsewhere) to focus on the strongest and most robust signals in each assay. Measurements with differential expression in both assays were considered concordant. We visualized the concordance between these assays with paired heatmaps displaying upregulated and downregulated measurements. We summarized these results with a Euler diagram showing set relationships between upregulated and downregulated measurements across all ligand treatments.

### Comparison of RNAseq and L1000 assays

To assess the concordance between gene expression profiles generated by both the RNAseq and L1000 assays, we first filtered Collection 1 Level 3 data from both datasets to contain only samples and transcripts directly measured by both assays, then z-transformed the filtered datasets. We calculated the Pearson’s correlation between the RNAseq and L1000 z-scores for all pairwise combinations of samples, then compared the distributions of treatment-matched and treatment-mismatched samples. Samples with the same ligand treatment and time point were considered treatment matched. We used a Mann-Whitney U test was used to test for differences in mean correlation between the treatment-matched and -mismatched groups.

### Comparison of ATACseq and RNAseq assays

To compare gene expression to chromatin accessibility at the respective transcriptional start site (TSS), we quantified chromatin accessibility using bedtools multiBamCov (v. 2.26.0) .[Chromatin accessibility was quantified in windows ±500 bp from TSS coordinates provided by the R package TxDb.Hsapiens.UCSC.hg38.knownGene [PMID 20110278]. The most-accessible TSS was selected for genes with multiple TSS. Integer counts were transformed using the variance-stabilized transformation from the R package DESeq2 (v. 1.24.0). Genes within the MHC region of chromosome 6 (chr6: 28510120-33480577) were excluded from this analysis; ATACseq data from this region had poor alignment due to alternative contigs for this region in the hg38 genome assembly. Median VST-transformed TSS accessibility was compared to median Level 3 RNAseq data for the EGF_48 condition.

We also compared the 10 most-variant ATACseq TF motifs (by standard deviation) to single-sample gene set enrichment scores computed for the same TFs from Level 3 RNAseq data, using the R package GSVA (v 1.32.0) and the TF-gene target mappings from the ReMap ChIP-seq library (as described above).

### Multi-omic module detection

To identify coordinately regulated multi-omic modules, we performed normalization, data scaling, feature selection and cluster analysis on molecular features induced by ligand treatments. For the GCP, RPPA and CyCIF datasets we used limma to normalize to CTRL_0, summarize across the replicates and calculate adjusted p-values using Benjamini-Hochberg correction; we used DESeq2 to analyze the RNAseq data in a similar manner. We used chromVAR to aggregate chromatin accessibility peaks that share common motifs and then the individual motif enrichment scores of transcription factor families. We applied the rrscale transformation to each assay data set to minimize differences in the assay-specific data distributions^70^. In brief, each assay’s T0 CTRL-normalized data was rrscaled independently with Box Cox negative and asinh transformations using an infinite z score cutoff. This transformation yields data matrices for each assay that have symmetrical Gaussian-shaped distributions, making them suitable for parametric statistics. We selected a subset of highly variant and biologically interpretable features from the 24H and 48H samples from each assay. In GCP and RPPA assays, features in the lowest variance quartile were removed. For the CyCIF, RNAseq, and GCP assays, features were retained if, for any condition, the absolute log fold change was greater than 1.5 and the p-value was less than 0.05. For the RPPA assay, we used a log fold-change threshold of 0.75 to account for differences in the RPPA data distribution. All ATACseq motif family scores were retained.

We performed k-means clustering using partitioning around medoids and a gap statistic analysis using the firstSEmax method to identify the optimal number of clusters (R package cluster, version 2.1.2). In brief, the gap statistic method runs PAM clustering on the integrated data matrix once for each k value, where k = 2:25. Then for each k, we performed PAM clustering on 100 randomized permutations of the data that have structure similar to the actual data. At each k, the gap is calculated as the difference in the log of the within-groups sum of squares of the actual versus randomized data. To cluster the features, we use partitioning around medoids (PAM) clustering for the optimal number of clusters defined in the previous step (k = 18), with seeds randomly selected from the dataset. We repeated this 100 times to form an ensemble of partitions, then calculated consensus clusters from the ensemble using a hard euclidean (HE) method and 5 internal runs. We repeated this entire procedure 25 times and then calculated a final consensus clustering with the HE method from these 25 consensus clusters. We further refined these clusters by identifying and collapsing highly correlated clusters. In brief, we calculated the mean expression of features in each cluster for each condition and then computed Pearson correlations between all pairs of clusters. Next, we then used the R hclust function and the dendextend cutree function on the distance matrix of the correlations to identify highly correlated clusters. This resulted in combining 4 pairs of clusters to yield a final set of 14 modules for further analysis.

### Consensus principal component analysis

To explore how our method compares against other published multiomics approaches^73^, we performed a Consensus Principal Component Analysis (CPCA) using the R package MoCluster^74^ and then compared the clusters to the refined molecular modules described above. In brief, the same features used in the consensus PAM clustering were input as separate blocks to the CPCA algorithm. For each Joint Latent Variable (JLV), the principal components of each assay (block) are calculated as the block latent variables (BLVs), normalized to 1, softly thresholded using a sparsity parameter (0.9) that controls the number of non-zero values and used to iteratively converge on a joint latent variable, which maximizes the correlation between the BLVs. Based on knee analysis of the CPCA pseudoeigenvalues, we kept the first 8 JLVs.

### Module TF enrichment analysis

We identified transcription factors enriched in the integrated modules by submitting all RNAseq features from each integrated module to the ChEA3 web-based transcription factor enrichment tool ChEA3^72^, which identifies transcription factors enriched for a list of genes using Fisher’s exact test. We limited our analyses to transcription factor targets in the ReMap ChIP-Seq library and considered transcription factors significantly enriched if the FDR-corrected q-value was less than 0.2.

### Module pathway enrichment analysis

To identify pathways enriched in each module, we used the Reactome pathway enrichment analysis tool (https://reactome.org/) to analyze the genes in each module. In brief, this analysis performs a binomial test of each gene set of 2516 curated pathways in the Reactome database. We identified significantly enriched pathways as those with FDR q-values (Benjamini-Hochberg method) < 0.2, gene ratios > 0.1, and pathways that included a minimum of 5 and maximum of 500 genes. To aid visual interpretation, only the top three pathways for each module sorted by FDR and descending gene ratio are shown in Fig. 5b.

### Module expression scores

To calculate the expression of modules across different samples in our MCF10A dataset, we computed the mean expression of features in each module. To assess expression of the modules in external datasets (e.g. GTEx), we focused on the RNAseq features in each module and computed their mean expression. For our analysis of Module 10 gene expression in a panel of breast cancer cell lines, we processed and aligned raw sequence data using the Docker-based RNA-seq pipeline^105^ described in **RNAseq pre-processing and QC**, then normalized the data using the variance-stabilizing transformation in the R package DESeq2^106^. We used a Mann-Whitney U test to test for differences in mean Module 10 gene expression between groups.

### Set analysis

Set analysis was used to identify features significantly induced by a single ligand (ligand-specific) or multiple ligands (shared). The input to the set analysis was the integrated and scaled matrix of log fold change values derived from the multi-omic module analysis. Each feature in the multi-omic matrix was labelled either ‘Unique’ or ‘Shared’. Features were defined as ‘Unique’ if they were significantly perturbed by only a single ligand, with log fold change greater than or equal to |1.5| and Benjamini-Hochberg adjusted p-value less than .05, relative to time 0. Features that were significantly regulated by two or more ligands were labelled ‘Shared.’

### Statistics and reproducibility

When testing for statistical significance, we adjusted for multiple testing using the Benjamini-Hochberg method. Assays were performed on samples in biological triplicate, as described in Cell Culture Methods. We used a threshold of q = 0.01 for individual analyses of assay datasets (RNAseq and RPPA) and q = 0.05 for phenotypic behavior comparisons to identify only the largest and most robust responses in the data, and a less stringent alpha of q = 0.2 for all other analyses. The significance of list-based enrichment analyses (CHEA3, L1000 FWD) was evaluated using Fisher’s exact tests. We used the nonparametric Mann-Whitney U test to test for between-group differences in RNA-L1000 correlation coefficients and Module 10 gene expression.

## Supplementary information

Supplementary Information

Description of Additional Supplementary Files

Supplementary Data 1

Supplementary Data 2

Supplementary Data 3

Supplementary Data 4

Supplementary Data 5

Supplementary Data 6

Supplementary Data 7

Supplementary Data 8

Supplementary Data 9

Supplementary Data 10

Supplementary Data 11

Supplementary Data 12

Supplementary Data 13

Supplementary Data 14

Supplementary Data 15

Supplementary Data 16

Supplementary Data 17

Supplementary Data 18

Supplementary Data 19

Supplementary Data 20

Supplementary Data 21

Supplementary Data 22

Supplementary Data 23

Supplementary Data 24

Supplementary Movie 1

Supplementary Movie 2

Supplementary Movie 3

Supplementary Movie 4

Supplementary Movie 5

Supplementary Movie 6

Supplementary Movie 7

Supplementary Movie 8

Supplementary Movie 9

## Data Availability

Data, metadata and additional analysis reports are available at: synapse.org/LINCS_MCF10A. Raw RNAseq and ATACseq data generated for this study can be accessed from the Gene Expression Omnibus (GSE152410). Datasets for figures are as follows: Fig. 2c–g in Supplementary Data 1; Fig. 2h in Supplementary Data 2, 3; Figs. 3a and 6b in Supplementary Data 4; Fig. 3d in Supplementary Data 6; Fig. 3f in Supplementary Data 7; Fig. 4a in Supplementary Data 8; Fig. 4c in Supplementary Data 12; Fig. 3b in Supplementary Data 5; Figs. 6c and 7c in Supplementary Data 17; Fig. 6d, e in Supplementary Data 16; Figs. 6g, h and 7b in Supplementary Data 15; Fig. 7e in Supplementary Data 20. Primary source data for Fig. 6g from GSE99116. Primary source data for Fig. 6h is hosted on Synapse.org with Synapse ID: syn2346643 (https://www.synapse.org/#!Synapse:syn2346643/wiki/232048). Supplementary Data 23 contains metadata for the experimental samples and can be merged with Level 3 data for each assay. All other data are available from the corresponding author on reasonable request.

## Code availability

Unless otherwise stated, analyses were performed in R (https://www.R-project.org). R packages used in analyses included: tidyverse^116^ (version 1.3.1), ComplexHeatmap (version2.8.0), httr (version 1.4.2) and rmarkdown (version 2.9). A complete list of packages and their versions can be found in analysis scripts available at https://github.com/MEP-LINCS/MDD. The DOI is https://zenodo.org/badge/latestdoi/189112490^117^. Supplementary Data 24 contains a mapping of figures and tables in this paper to the scripts that created them.
