# Supplementary material for: A multi-omic analysis of MCF10A cells provides a resource for integrative assessment of ligand-mediated molecular and phenotypic responses
Source: Commun Biol. 2022 Oct 7;5:1066. doi: 10.1038/s42003-022-03975-9 (PMC9546880; doi:10.1038/s42003-022-03975-9)
Supplement: Supplementary file 1 — Supplementary Information [file 42003_2022_3975_MOESM1_ESM.pdf]

SUPPLEMENTARY INFORMATION

A multi-omic analysis of MCF10A cells provides a resource for integrative assessment of ligand-mediated molecular and phenotypic responses

Sean M. Gross[1], Mark A. Dane[1], Rebecca L. Smith[1], Kaylyn L. Devlin[1], Ian McLean[1], Daniel S. Derrick[1], Caitlin E. Mills[2], Kartik Subramanian[2], Alexandra B. London[3], Denis Torre[3], John Erol Evangelista[3], Daniel J.B. Clarke[3], Zhuorui Xie[3], Cemal Erdem[4], Nicholas Lyons[5], Ted Natoli[5], Sarah Pessa[5], Xiaodong Lu[5], James Mullahoo[5], Jonathan Li[6], Miriam Adam[6], Brook Wassie[6], Moqing Liu[1], David F. Kilburn[1], Tiera A. Liby[1], Elmar Bucher[1], Crystal Sanchez-Aguila[1], Kenneth Daily[7], Larsson Omberg[7], Yunguan Wang[2], Connor Jacobson[2], Clarence Yapp[2], Mirra Chung[2], Dusica Vidovic[8,9,10], Yiling Lu[11], Stephan Schurer[8,9,10], Albert Lee[12], Ajay Pillai[13], Aravind Subramanian[5], Malvina Papanastasiou[5], Ernest Fraenkel[5,6], Heidi S. Feiler[1], Gordon B. Mills[14,15], Jake Jaffe[5], Avi Ma'ayan[3], Marc R. Birtwistle[4], Peter K. Sorger[2], James E. Korkola[1,15], Joe W. Gray[1,15], Laura M. Heiser[1,15,*]

[1]Department of Biomedical Engineering, OHSU, Portland, OR, USA
[2]Laboratory of Systems Pharmacology, Department of Systems Biology, Harvard Program in Therapeutic Science, Harvard Medical School, Boston, MA, USA
[3]Department of Pharmacological Sciences, Mount Sinai Center for Bioinformatics, Icahn School of Medicine at Mount Sinai, New York, NY, USA
[4]Department of Chemical and Biomolecular Engineering, Clemson University, Clemson, SC, USA
[5]Broad Institute of MIT and Harvard, Cambridge, MA, USA
[6]Department of Biological Engineering, Massachusetts Institute of Technology, Cambridge, MA, USA
[7]Sage Bionetworks, Seattle, WA, USA
[8]Sylvester Comprehensive Cancer Center, University of Miami, FL 33136, USA
[9]Department of Molecular and Cellular Pharmacology, Miller School of Medicine, University of Miami, Miami, FL 33136, USA
[10]Institute for Data Science & Computing, University of Miami, FL 33136, USA
[11]Department of Genomic Medicine, Division of Cancer Medicine, The University of Texas MD Anderson Cancer Center, Houston, TX, USA
[12]Heart, Lung, and Blood Institute, National Institutes of Health, Bethesda, USA
[13]Human Genome Research Institute, National Institutes of Health, Bethesda, USA
[14]Division of Oncological Sciences, OHSU, Portland, OR, USA
[15]Knight Cancer Institute, OHSU, Portland, OR, USA
*Correspondence: heiserl@ohsu.edu ORCID: 0000-0003-3330-0950

Key words: perturbation, phenotype, integrative analysis, module, LINCS, multi-omic

Supplementary Figure 1. Experimental and bioinformatic approaches to identify high impact ligands

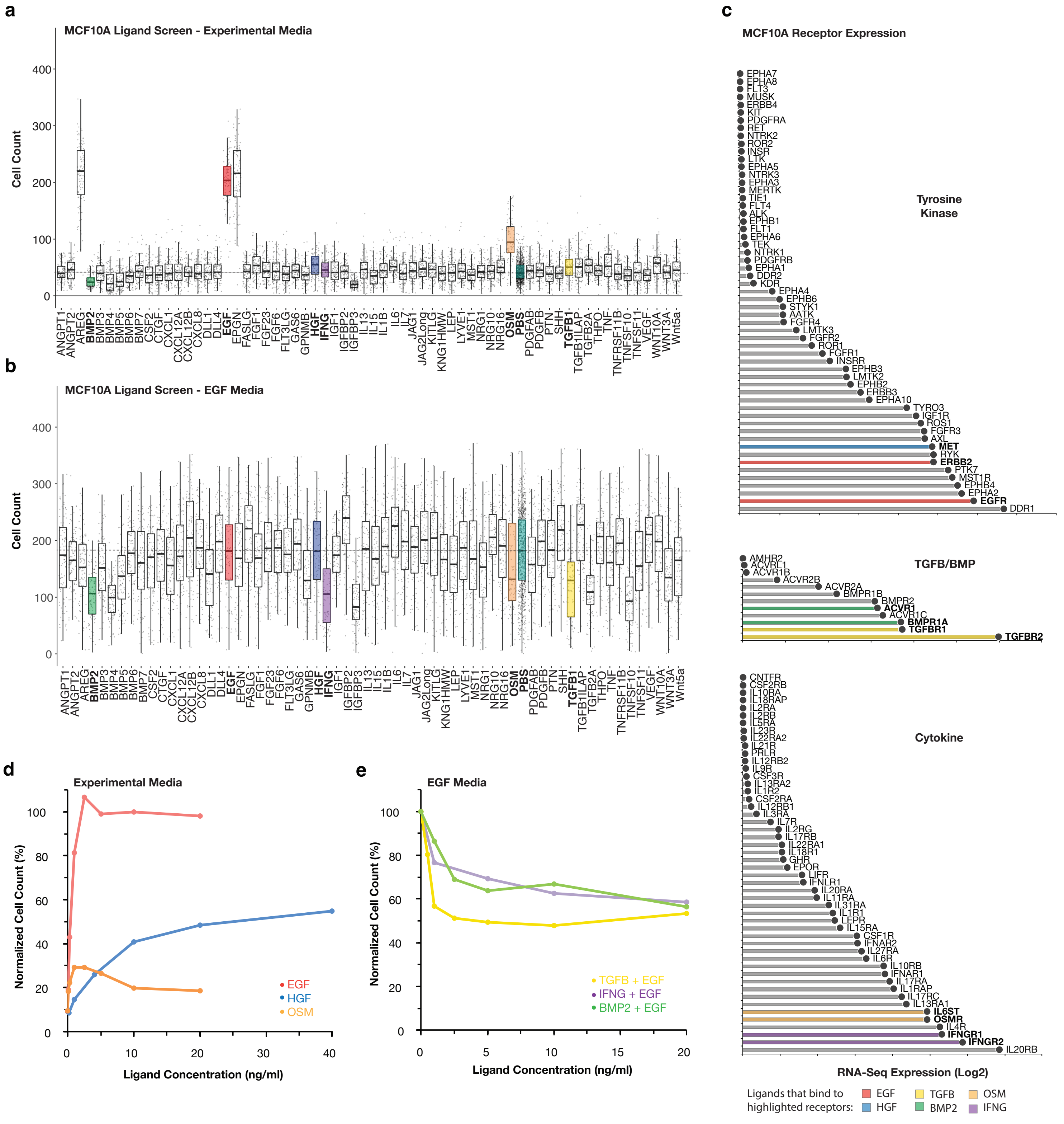

a Microenvironmental assay (MEMA) to identify ligands that modulate MCF10A cell numbers. Cells were treated with ligands in experimental media lacking EGF and cell numbers were counted after 72H. In the boxplots, lower and upper hinges correspond to the first and third quartiles. The median is shown as the center line. Whiskers extend to no further than +/- 1.5 * IQR from the hinge, where IQR is the inter-quartile range, or distance between the first and third quartiles. **b** MEMA assay results for MCF10A cells treated with ligands in experimental media containing EGF. Boxplots draw as described in a. **c** MCF10A transcript expression from three receptor classes: Tyrosine kinase, cytokine, and TGFB/BMP. Transcript values are drawn from RNAseq measures for untreated cells in exponential growth. The primary receptors for the six ligands are highlighted HGF: MET (Blue), EGF:EGFR/ERBB2 (Red), BMP2:BMPR1B/BMPR1A (Green), TGFB: TGFBR1/TGFBR2 (Yellow), OSM: IL6ST/OS-MR (Orange), and IFNG:IFNGR1/IFNGR2 (Purple). **d** Cell count dose-responses after treatment with EGF, HGF, and OSM. Cell counts at 72H were normalized to the 10 ng/ml EGF condition. **e** Cell count dose responses for TGFB1, IFNG, and BMP2. Each of the ligands were supplement-ed with 10 ng/ml EGF. Cells counts at 72H were normalized to the EGF condition with no secondary ligand.

Supplementary Figure 2. Comparison of ligand and small molecule inhibitor signatures

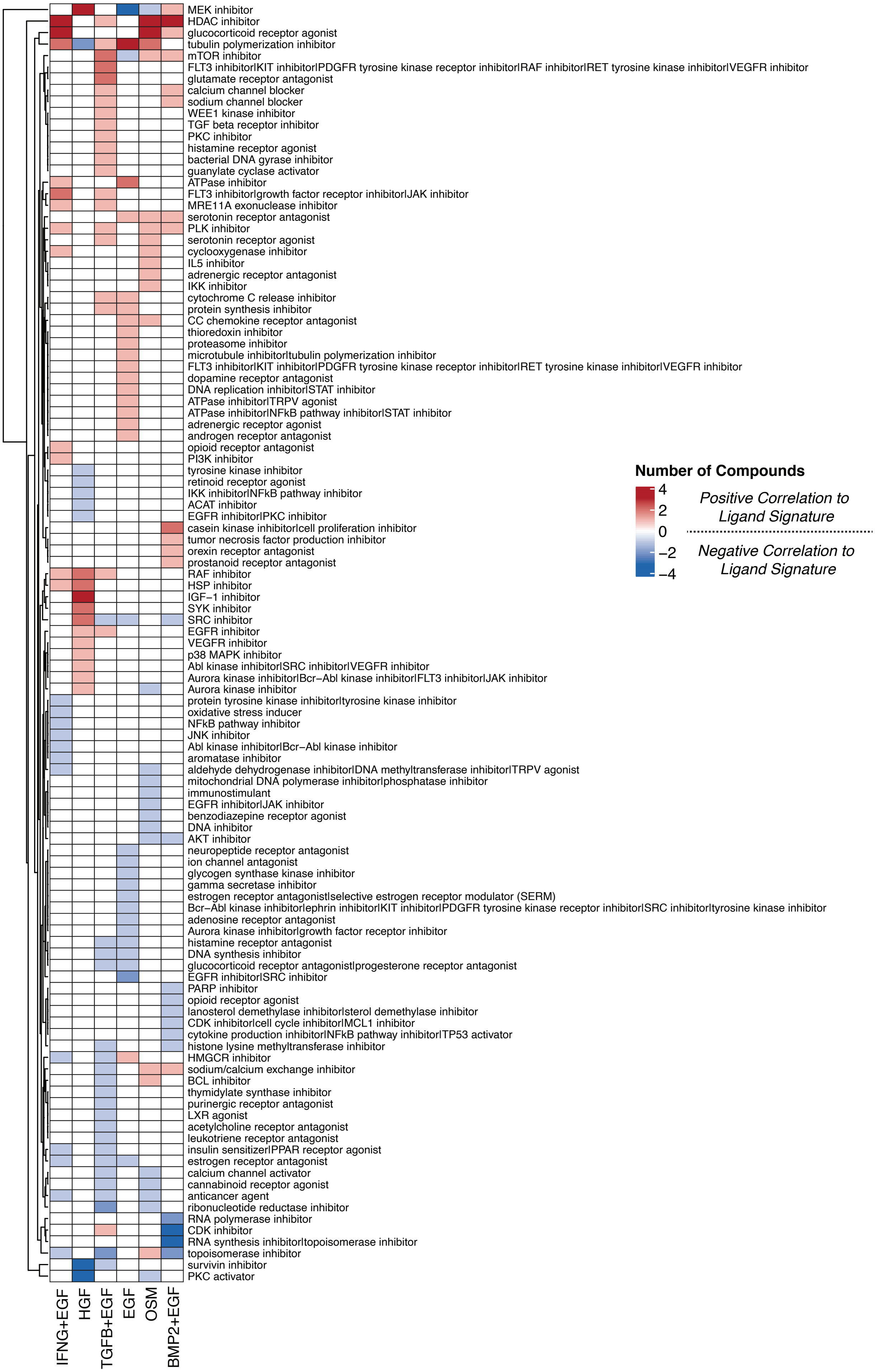

Supplementary Figure 2. Comparison of ligand and small molecule inhibitor signatures

We leveraged the LINCS L1000 database of drug response signatures to identify targeted inhibitors that are shared by each ligand signature. Heatmap represents the number of compounds that have correlated (red) or anti-correlated (blue) signatures with each ligand (Fisher exact test, q-value < 0.2). The ligand panel activated many of the same signatures as small molecule inhibitors, indicating that shared molecular responses can be elicited by these distinct perturbagen classes.

Supplementary Figure 3. IFNG responses are dynamically encoded across multiple molecular modalities

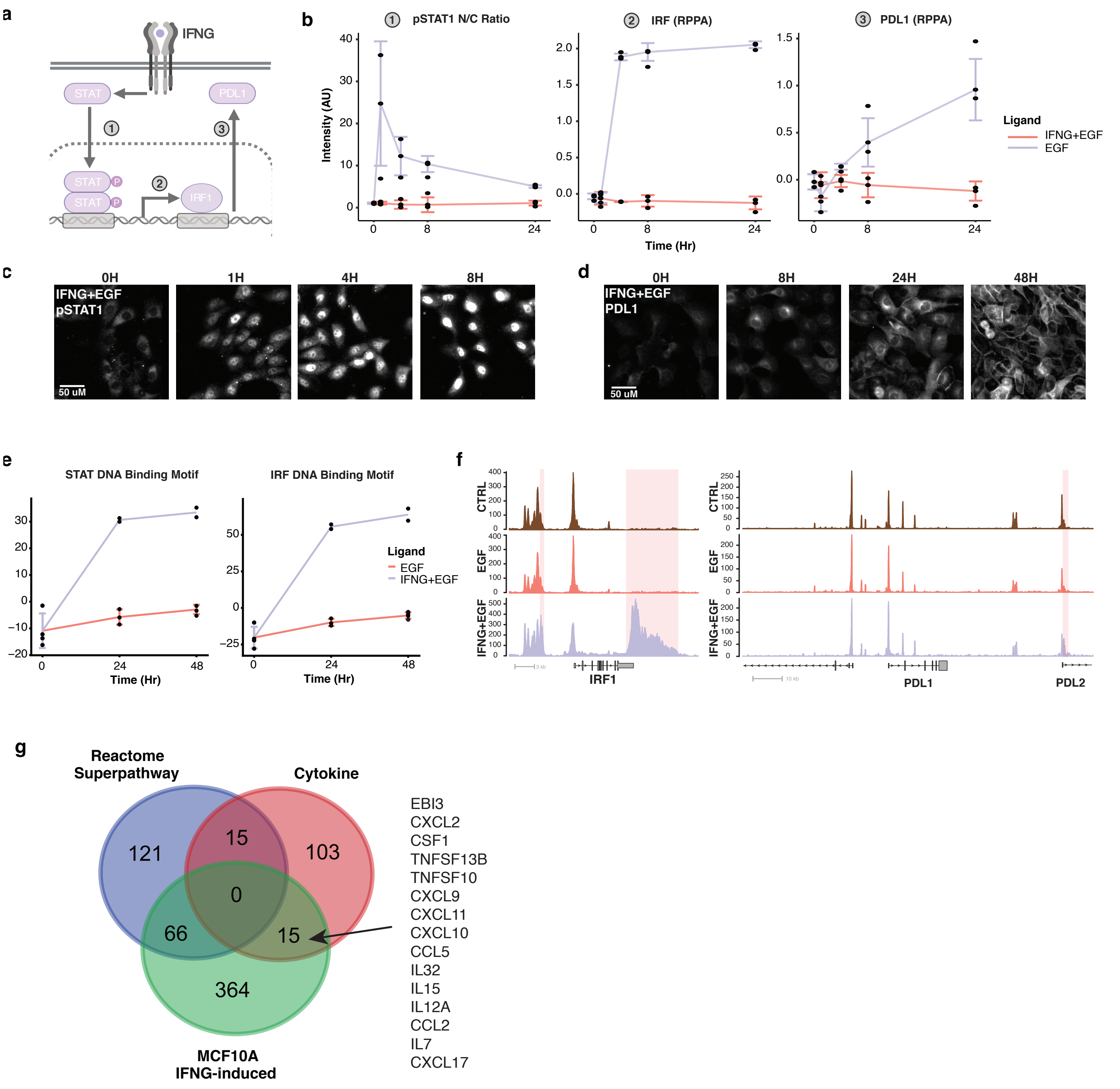

a Cartoon of canonical STAT pathway activation after treatment with IFNG ligand. **b** Line graphs show induction of pSTAT1, IRF1 and PDL1 protein expression following IFNG treatment, as measured by CyCIF and RPPA assays. Error bars represent the standard deviation from the median value. Values from each replicate are plotted as individual data points. **c,d** Cyclic immunofluorescence images show changes in STAT1 and PDL1 protein abundance and localization induced by IFNG+EGF treatment. **e** Line graphs show enrichment of STAT-family and IRF-family motifs inferred from ATACseq chromatin accessibility data for IFNG+EGF and EGF conditions. Error bars represent standard deviation. Low quality EGF+IFNG samples were removed, yielding only two replicates for this treatment. **f** Chromatin accessibility near the IRF1 (chr5: 132390440-191020960) and PDL1 (chr2: 190908460-191020960) gene loci. The local gene region for IRF1 showed a new peak in the promoter region and a large accessibility change in the 3' region. IFNG did not induce new ATACseq peaks in PDL1, however IFNG induced a new peak in the adjacent PDL2 gene (PDCD1LG2). DNA regions with changes in accessibility are marked with a red background. g Venn diagram showing the overlap between the Reactome IFNG pathway, curated cytokine gene lists and, and genes induced by IFNG+EGF treatment. The 15 cytokines induced by IFNG+EGF are listed on the right.

Supplementary Figure 4. Comparison across assays reveals concordance in response to ligand treatment

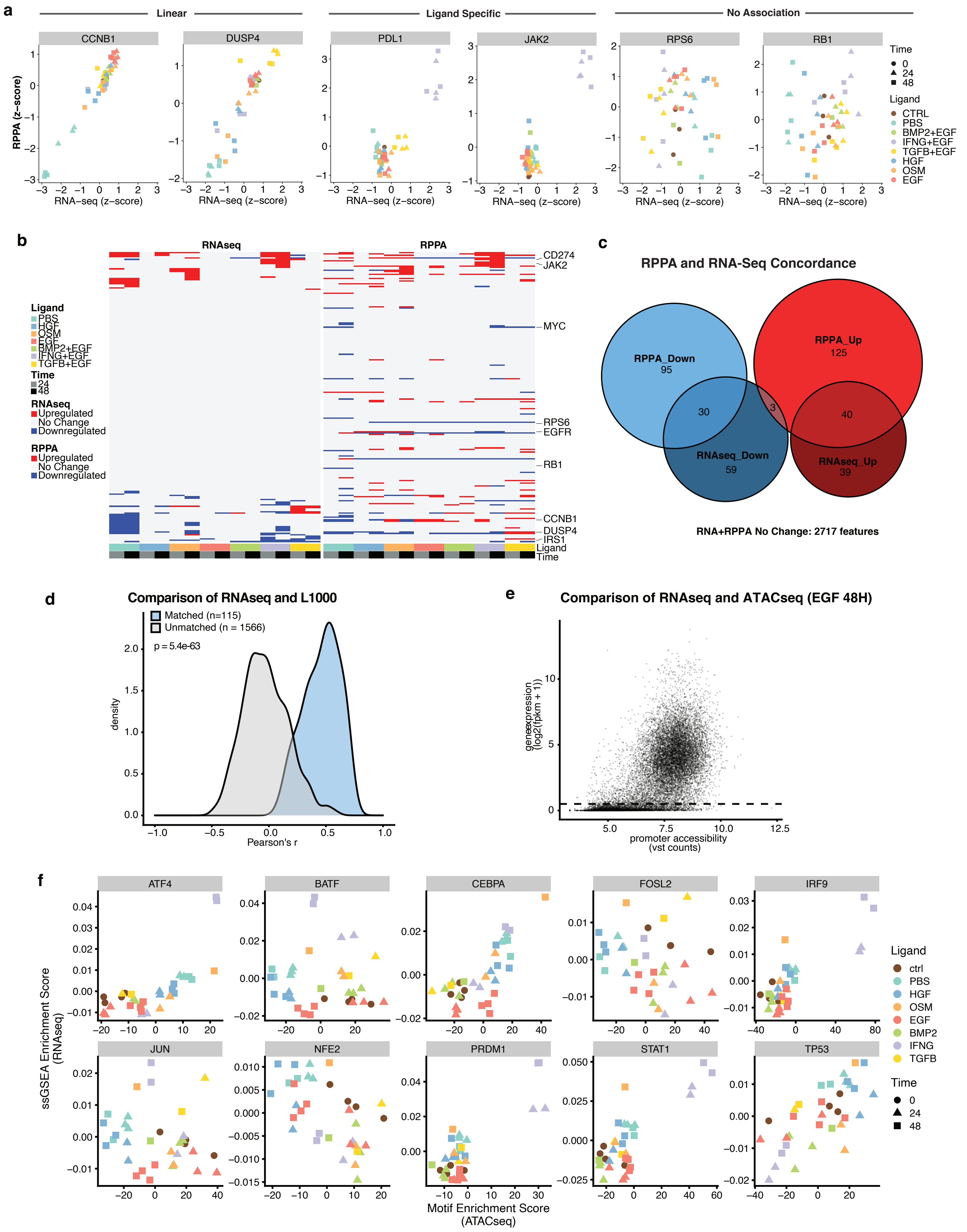

Supplementary Figure 4. Comparison across assays reveals concordance in response to ligand treatment

a Scatter plots of paired RPPA and RNAseq measurements, showing three classes of observed relationships: linear, ligand-specific, and no change. **b** Heatmaps show genes and proteins with significantly up- or down-regulated expression after ligand treatment. Genes were defined as significantly up- or down-regulated from RNAseq (q-value ≤ 0.01; log2FC ≥ |1.5|), while proteins were defined from analysis of RPPA (q-value ≤ 0.01; log2FC ≥ |0.5|). **c** Euler diagram showing intersections of differentially expressed RPPA proteins and RNAseq genes. The majority of features that were induced in both assays showed concordant responses, defined as both modalities induced in the same direction. **d** Distributions of Pearson's correlation coefficients from RNAseq and L1000 gene expression profiles of treatment-matched and treatment-mismatched samples. Both datasets were z-transformed after filtering to overlapping genes and samples. Transcriptional profiles from treatment-matched samples were significantly more correlated than were transcriptional profiles from nonmatched samples. **e** Dot plot showing the relationship between ATACseq transcriptional start site (TSS) accessibility and gene expression in the EGF 48H samples. Note the switch-like relationship between gene expression and accessibility at the TSS, as has been described previously. The horizontal dotted line indicates the threshold for a gene defined as being expressed. **f** Comparison of ATACseq and RNAseq data for the 10 most variant transcription factor motifs (by standard deviation). Motif enrichment scores (x axis) represent the deviation in motif chromatin accessibility from an expectation based on the average accessibility of the motif across all samples, while ssGSEA scores (y axis) represent the degree of coordinated expression of the TF target gene expression.

Supplementary Figure 5. Integrated analysis methods.

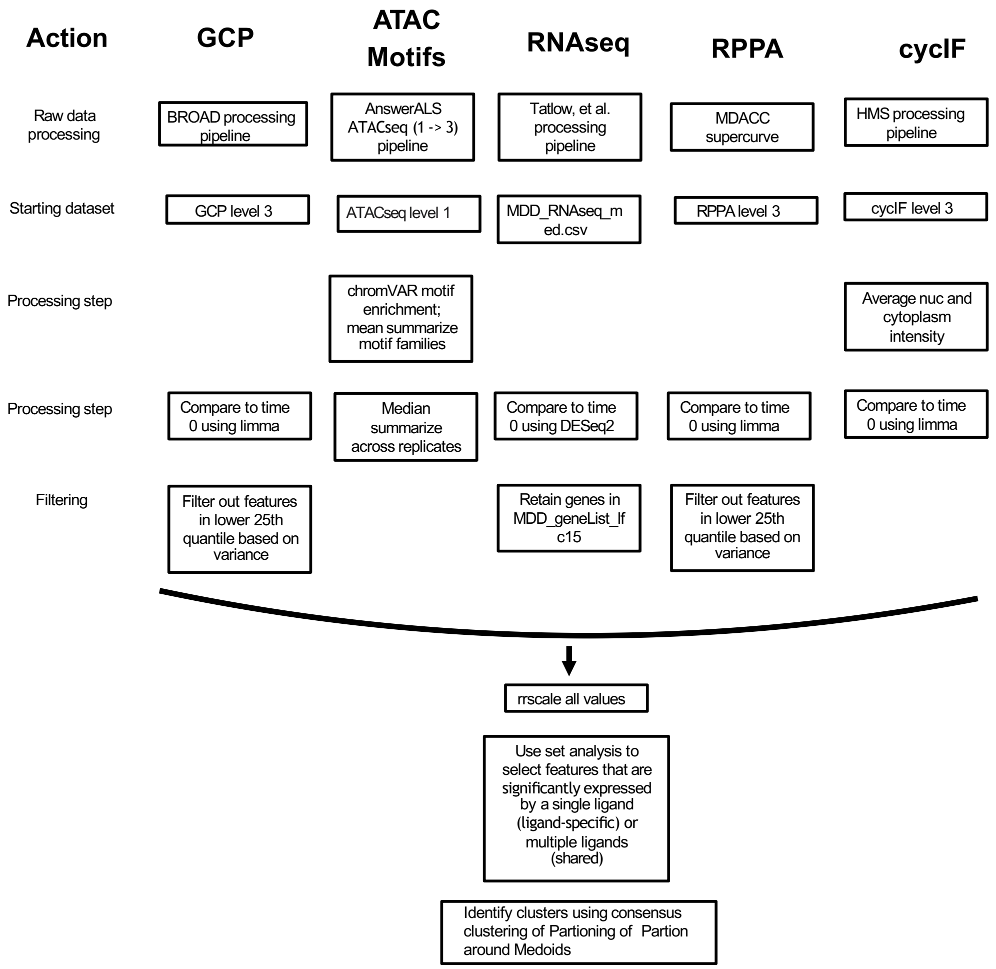

Pre-processed data from each assay are summarized, filtered, and scaled before being combined into a single matrix. PAM clustering with gap analysis was used to identify the optimal number of clusters represented in the integrated data matrix, which resulted in 18 modules. Pearson correlation analysis was used to identify pairs of clusters that showed similar expression patterns; two pairs of modules were combined to yield a final set of 14 modules.

Supplementary Figure 6. Identification and characterization of integrative molecular modules

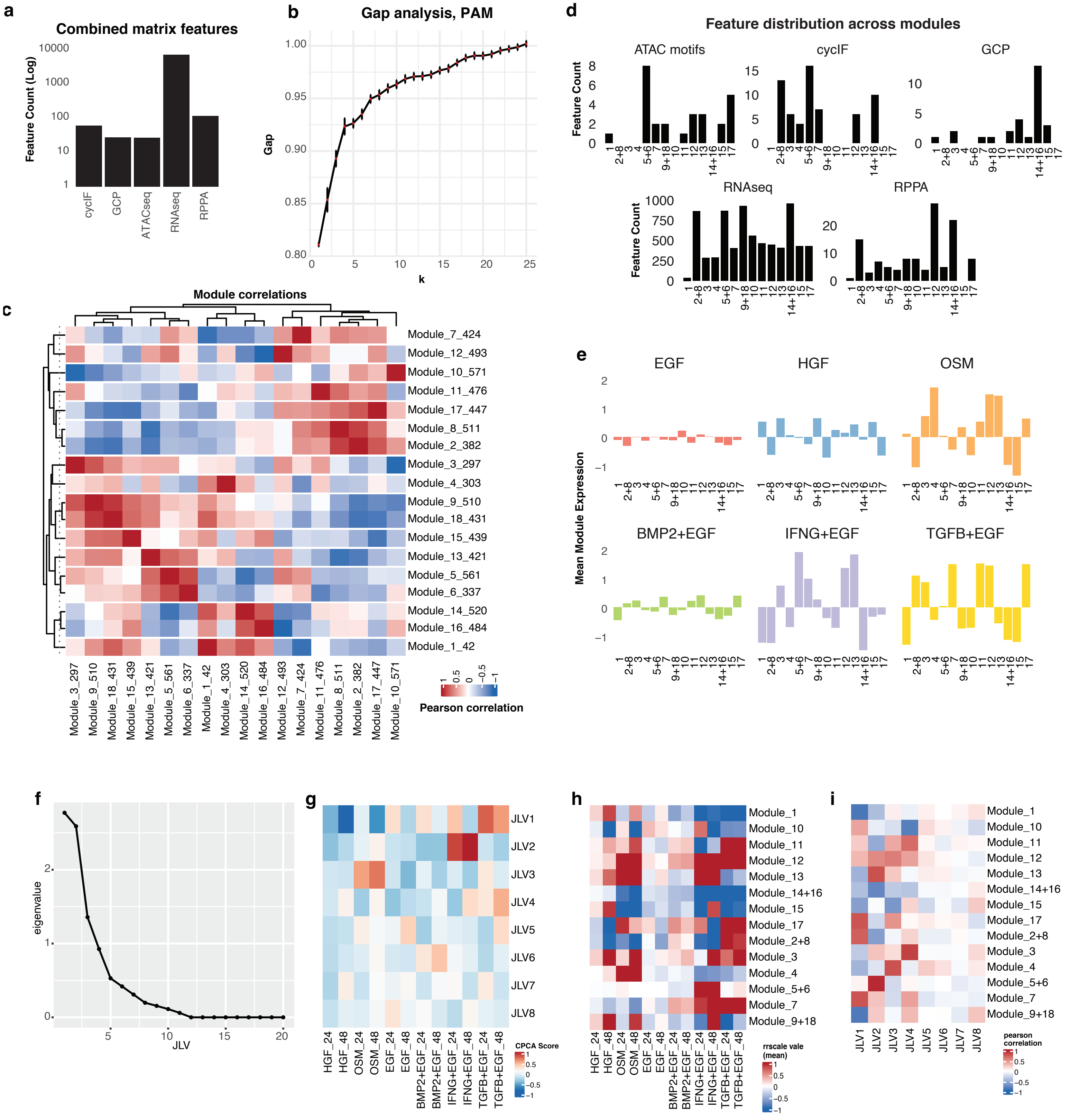

a Bar plot showing the number of features for each assay included in the integrative modules; note log 10 scale. **b** Gap analysis used to identify the optimal number of modules. Error bars show +/- the standard error from the simulations. **c** Module correlation matrix showing Pearson correlation values. Highly correlated cluster pairs 2+8, 5+6, 9+18 and 14+16 were combined to yield 14 clusters. **d** Bar plot showing the distribution of features for each assay across modules. **e** Bar plot showing the mean module expression for each of the ligand treatments. **f** Consensus Principal Component Analysis identifies multiomic modules, and analysis of the resultant eigenvalues for the joint latent variables (JVLs) shows a knee at 8 modules. **g** Heatmap showing expression of the top 8 JLVs for each treatment condition. Heat color indicates CPCA score. **h** Heatmap showing the mean expression of the optimized modules across the treatment conditions. **i** Heatmap showing the correlation in expression patterns for JLVs and module scores shown in g and h. There is high correlation between the first 4 JLVs and the consensus module scores.

Supplementary Figure 7. GTEx expression analysis

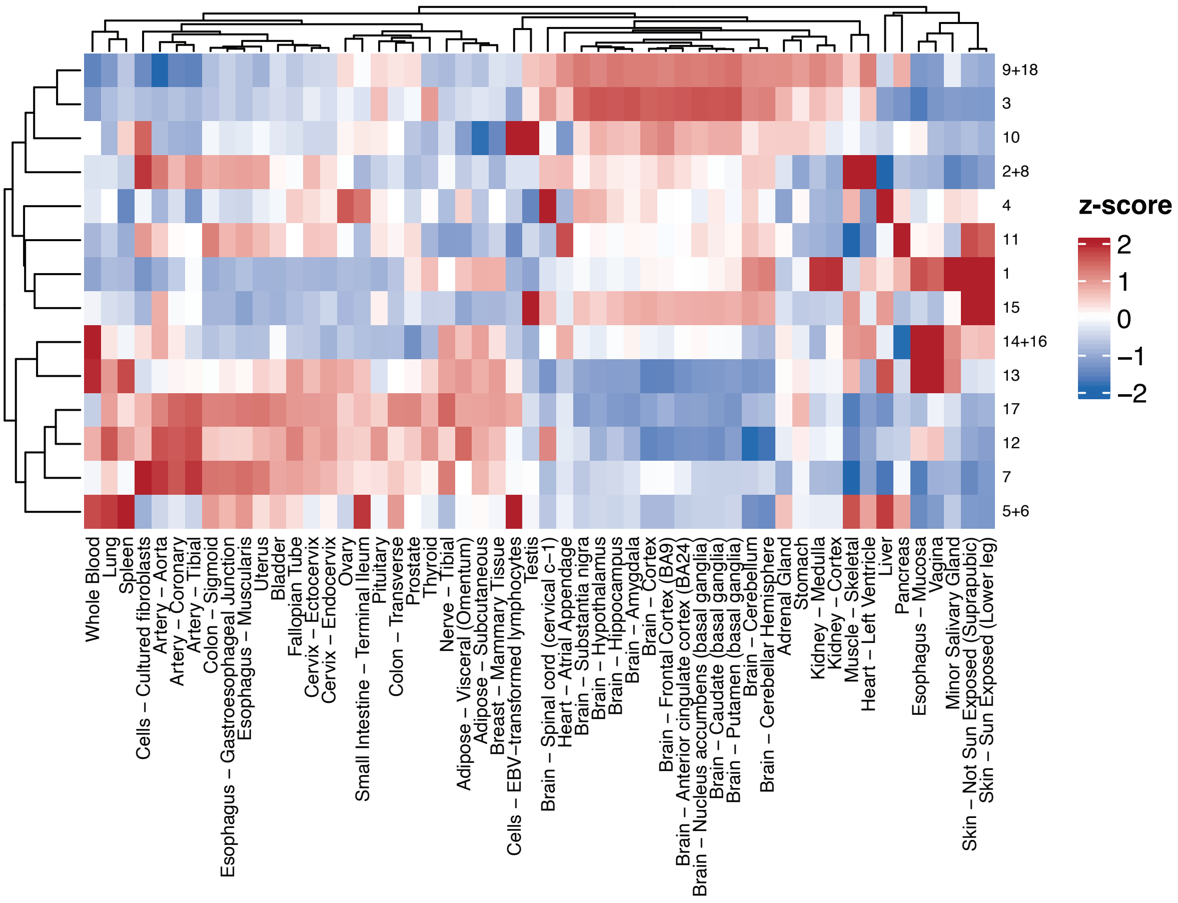

Heatmap showing GTEX tissue expression of the 14 integrative molecular modules reveals tissue-specific expression, suggesting molecular programs that may be particularly important for mediating normal and diseased functions across tissues.

Descriptions of Supplementary Data and Movie files

Synapse data resource
Raw and processed data and their metadata are available for free download at synapse.org/LINCS_MCF10A where the computational methods are described along with links to the code.

Supplementary Movies 1-7
Live cell imaging of MCF10A cells grown in experimental media and treated at 0H with EGF, HGF, OSM, TGFB+EGF, IFNG+EGF, BMP2+TGFB, or PBS. Images were collected every 30 min for 48 hours (FLR IncuCyte, 10X objective).

Supplementary Movies 8-9
Live cell imaging of MCF10A cells grown in experimental media and treated at 0H with OSM (Movie 8), or OSM+Ruxolitinib (Movie 9). Images were collected every 30 min for 48 hours (IncuCyte, 10X objective).

Supplementary Data 1
Source data for Figures 2c-g. Cell phenotype data gathered from four-channel immunofluorescence imaging, filtered to the 0 and 48 hour time points and median summarized at the well level. A description of the methods used to calculate each phenotype metric is described in Methods. Source data are available at https://www.synapse.org/#!Synapse:syn13363381.

Supplementary Data 2
Source data for Figure 2h. Cell migration data from 48 hour live cell imaging after ligand treatment. These data are combined with Supplementary Data 3 to show the migration and mitotic events from an equal number of 48 hour lineages from each treatment. These data and that in Supplementary Data 3 were collected using the ImageJ MtrackJ plugin and the output is described at https://imagescience.org/meijering/software/mtrackj/manual/.

Supplementary Data 3
Source data for Figure 2h. Miotic events from 48 hour live cell imaging as measured using the ImageJ plugin MtrackJ.

Supplementary Data 4
Source data for Figure 3a and 6b. Reverse Phase Protein Array data for six time points and seven ligand treatments. The data is from three replicates that are median summarized. The figures show data from antibodies whose rows were selected from the antibody column. All lines start at the antibody's time point 0 value represented as ctrl_0.

Supplementary Data 5
Source data for Figures 3b. Data from three replicates of Reverse Phase Protein Array data for six time points and seven ligand treatments. Metadata for the columns are in Supplementary Data 23 and are explained at https://www.synapse.org/#!Synapse:syn12979102.

Supplementary Data 6
Source data for Figure 3d. Variance for the first seven principal components of the RPPA assay computed with Measuring Association between VaRIance and Covariates (MAVRIC) method. The experimental covariates are time, replicate and ligand. The category 'Discarded' refers to

variance contained within principal components that have an eigenvalue less than 0.7, which were discarded at the outset of the analysis.

Supplementary Data 7
Source data for Figure 3f. MAVRIC variances for all assays. The fractional variance explained by experimental covariates of time, replicate and ligand. The experimental covariates are time, replicate and ligand. The category ‘Discarded’ refers to variance contained within principal components that have an eigenvalue less than 0.7, which were discarded at the outset of the analysis.

Supplementary Data 8
Source data for Figure 4a. The number of molecular features perturbed by one or more ligand treatment. Features that are considered shared features are significantly modulated by two or more ligands as compared to time 0, which required a log fold change greater than 1.5 and P-value less than 0.05. Unique features are significantly modulated by only a single ligand.

Supplementary Data 9
Data for the shared molecular features that are significantly modulated by two or more ligands with a log fold change greater than 1.5 and P-value less than 0.05 relative to time 0.

Supplementary Data 10
Data for the unique molecular features that are significantly modulated by only one ligand with a log fold change greater than 1.5 and P-value less than 0.05 relative to time 0.

Supplementary Data 11
Source data for Figure 4b. Cross correlation values computed from 24 and 48H time points across assays.

Supplementary Data 12
Source data for Figure 4c. ATACseq transcription factor motif values identified as positively enriched.

Supplementary Data 13
Source data for Figure 4d. MSigDB Hallmark Pathways gene set enrichment scores computed from RNAseq data at 24 hours.

Supplementary Data 14
Source data for Supplementary Figure 2**.** Drug signatures from the L1000FWD library that are most similar or dissimilar to the RNA-seq responses of the ligand treatments compared to time 0.

Supplementary Data 15
Source data for Figures 6g, 6h and 7b. All MCF10A Integrative Molecular Modules features’ normalized (rrscaled) values annotated with their cluster assignment, assay type, time point and set designation.

Supplementary Data 16
Source data for Figure 6d and 6e. The following columns are used in this analysis of the Reactome Pathways Enrichments:
Cluster: multiomics module label

stId: Reactome stable identifier
name: Reactome pathway name
total: number of genes in the Reactome pathway
found: number of genes in the multiomics module that are in the Reactome pathway
fdr: fdr-corrected p-values for the multiomics genes found in the Reactome pathway
geneRatio: found/total

Filtering parameters used for Figure 6e are fdr <= 0.2, geneRatio >=0.05 and 10<total<500. The top three pathways for each module that meet the filtering criteria are displayed in the dotplot along with pathways selected by other modules.

Supplementary Data 17
Source data for Figure 6c and 7c. Transcription factor enrichment scores and statistics identified with ChEA3 analysis.

Supplementary Data 18
Source data for Supplementary Figure 7. Mean expression of RNAseq features from multi-omic modules computed for GTEx RNAseq data.

Supplementary Data 19
Source data for figure 6a. Additional annotations for RNA-seq features in Module 10.

Supplementary Data 20
Source data for Figure 7e. Multi-omic Module 4 BioPlanet enriched pathway values.

Supplementary Data 21
Metadata for the antibodies used in the CyCIF assay.

Supplementary Data 22
Quantitated image features from the CyCIF dataset.

Supplementary Data 23
Metadata file with annotations for all samples in all assays. Column definitions and usage are explained at https://www.synapse.org/#!Synapse:syn12979102.

Supplementary Data 24
Mapping of figures to scripts and datasets in the manuscript.
