# Supplementary material for: A multi-omic analysis of MCF10A cells provides a resource for integrative assessment of ligand-mediated molecular and phenotypic responses
Source: Commun Biol. 2022 Oct 7;5:1066. doi: 10.1038/s42003-022-03975-9 (PMC9546880; doi:10.1038/s42003-022-03975-9)
Supplement: Supplementary file 2 — Description of Additional Supplementary Files [file 42003_2022_3975_MOESM2_ESM.pdf]

File Name: Supplementary Data 1
Description: Source data for Figures 2c-g. Cell phenotype data gathered from four-channel immunofluorescence imaging, filtered to the 0 and 48 hour time points and median summarized at the well level. A description of the methods used to calculate each phenotype metric is described in Methods. Source data is available at https://www.synapse.org/#!Synapse:syn13363381.

File Name: Supplementary Data 2
Description: Source data for Figure 2h. Cell migration data from 48 hour live cell imaging after ligand treatment. These data are combined with Supplementary Data 3 to show the migration and mitotic events from an equal number of 48 hour lineages from each treatment. These data and that in Supplementary Data 3 were collected using the ImageJ MtrackJ plugin and the output is described at https://imagescience.org/meijering/software/mtrackj/manual/.

File Name: Supplementary Data 3
Description: Source data for Figure 2h. Miotic events from 48 hour live cell imaging as measured using the ImageJ plugin MtrackJ.

File Name: Supplementary Data 4
Description: Source data for Figure 3a and 6b. Reverse Phase Protein Array data for six time points and seven ligand treatments. The data is from three replicates that are median summarized. The figures show data from antibodies whose rows were selected from the antibody column. All lines start at the antibody's time point 0 value represented as ctrl_0.

File Name: Supplementary Data 5
Description: Source data for Figures 3b. Data from three replicates of Reverse Phase Protein Array data for six time points and seven ligand treatments. Metadata for the columns are in Supplementary Data 23 and are explained at https://www.synapse.org/#!Synapse:syn12979102.

File Name: Supplementary Data 6
Description: Source data for Figure 3d. Variance for the first seven principal components of the RPPA assay computed with Measuring Association between VaRIance and Covariates (MAVRIC) method. The experimental covariates are time, replicate and ligand. The category 'Discarded' refers to variance contained within principal components that have an eigenvalue less than 0.7, which were discarded at the outset of the analysis.

File Name: Supplementary Data 7
Description: Source data for Figure 3f. MAVRIC variances for all assays. The fractional variance explained by experimental covariates of time, replicate and ligand. The experimental covariates are time, replicate and ligand. The category 'Discarded' refers to variance contained within principal components that have an eigenvalue less than 0.7, which were discarded at the outset of the analysis.

File Name: Supplementary Data 8
Description: Source data for Figure 4a. The number of molecular features perturbed by one or more ligand treatment. Features that are considered shared features are significantly modulated by two or more ligands as compared to time 0, which required a log fold change greater than 1.5 and P-value less than 0.05. Unique features are significantly modulated by only a single ligand.

File Name: Supplementary Data 9
Description: Data for the shared molecular features that are significantly modulated by two or more ligands with a log fold change greater than 1.5 and P-value less than 0.05 relative to time 0.

File Name: Supplementary Data 10
Description: Data for the unique molecular features that are significantly modulated by only one ligand with a log fold change greater than 1.5 and P-value less than 0.05 relative to time 0.

Description: File Name: Supplementary Data 11
Source data for Figure 4b. 24 and 48 hour cross correlation values of ligand treatment molecular responses across the assays.

File Name: Supplementary Data 12
Description: Source data for Figure 4c. ATACseq transcription factor motif values identified as positively enriched.

File Name: Supplementary Data 13
Description: Source data for Figure 4d. MSigDB Hallmark Pathways gene set enrichment scores computed from RNAseq data at 24 hours.

File Name: Supplementary Data 14
Description: Source data for Supplementary Figure 2**.** Drug signatures from the L1000FWD library that are most similar or dissimilar to the RNA-seq responses of the ligand treatments compared to time 0.

File Name: Supplementary Data 15
Description: Source data for Figures 6g, 6h and 7b. All MCF10A Integrative Molecular Modules features' normalized (rrscaled) values annotated with their cluster assignment, assay type, time point and set designation.

File Name: Supplementary Data 16
Description: Source data for Figure 6d and 6e. The following columns are used in this analysis of the Reactome Pathways Enrichments:
Cluster – The multiomics module label
stId – Reactome stable identifier
name – Reactome pathway name
total – the number of genes in the Reactome pathway
found – the number of genes in the multiomics module that are in the Reactome pathway
fdr – fdr-corrected p-values for the multiomics genes found in the Reactome pathway
geneRatio – found/total

Filtering parameters used for Figure 6e are fdr <= 0.2, geneRatio >=0.05 and 10<total<500. The top three pathways for each module that meet the filtering criteria are displayed in the dotplot along with pathways selected by other modules.

File Name: Supplementary Data 17
Description: Source data for Figure 6c and 7c. Transcription factor enrichment scores and statistics identified with ChEA3 analysis.

File Name: Supplementary Data 18
Description: Source data for Supplementary Figure 7. Mean expression of RNAseq features from multi-omic modules computed for GTEx RNAseq data.

File Name: Supplementary Data 19
Description: Source data for figure 6a. Additional annotations for RNA-seq features in Module 10.

File Name: Supplementary Data 20
Description: Source data for Figure 7e. Multi-omic Module 4 BioPlanet enriched pathway values.

File Name: Supplementary Data 21
Description: Metadata for the antibodies used in the CyCIF assay.

File Name: Supplementary Data 22
Description: Quantitated image features included from the CyCIF dataset.

File Name: Supplementary Data 23
Description: Metadata file with annotations for all samples in all assays. Column definitions and usage are explained at https://www.synapse.org/#!Synapse:syn12979102.

File Name: Supplementary Data 24
Description: Mapping of figures to scripts and datasets in the manuscript.
